# Episiotomy and postpartum haemorrhage in women with moderate or severe anaemia: a cohort analysis of data from the WOMAN-2 trial

**DOI:** 10.1016/S2214-109X(25)00449-8

**Published:** 2026-02-01

**Authors:** The WOMAN-2 Trial Collaborators, Writing Committee, Judith Lieber, Folasade Adenike Bello, Rizwana Chaudhri, Kiran Javaid, Aasia Kayani, Mwansa Ketty Lubeya, Projestine Muganyizi, Oladapo Olayemi, Bellington Vwalika, Monica Arribas, Maha Asad, Eni Balogun, Amy Brenner, Amber Geer, Katharine Ker, Raoul Mansukhani, Henry Ochola, Danielle Prowse, John Tazare, Ian Roberts, Haleema Shakur-Still, WOMAN-2 Trial Steering Committee, France Donnay, Racheal Phiri, Tina Lavender, Ghazala Mahmud, Haleema Shakur-Still, Ian Roberts, Jens Kieckbusch, James Lawrence, Sari Watson, Data Monitoring Committee, Jane Armitage, Maria Quigley, Olufemi T Oladapo, Protocol Committee, Sabaratnam Arulkumaran, Imelda Bates, Rizwana Chaudhri, Bukola Fawole, Katharine Ker, Ian Roberts, Haleema Shakur-Still, Central coordinating team, Eni Balogun, Danielle Prowse, Danielle Beaumont, Charlotte Fleming, Monica Arribas, Collette Barrow, Llion Roberts, Amber Geer, Julio Gil Onandia, Madeleine Cargill, Andrew Thayne, Christiana Alao, Petula Buck, Raoul Mansukhani, Tim Collier, Katharine Ker, Mbwana Mohammed, Amy Brenner, Myriam Benyahia, Judith Lieber, Haleema Shakur-Still, Ian Roberts, Pakistan coordinating team, Rizwana Chaudhri, Aasia Kayani, Kiran Javaid, Tanzania coordinating team, Projestine Muganyizi, Alice Kawala, Rose Temba, Nigeria coordinating team, Oladapo Olayemi, Folasade Adenike Bello, Olujide Okunade, Zambia National coordinator, Bellington Vwalika

**Affiliations:** Clinical Trials Unit, London School of Hygiene and Tropical Medicine; Global Institute of Human Development, Shifa Tameer-e-Millat University, Islamabad, Pakistan; Muhimbili University of Health and Allied Sciences, Dar es Salaam, Tanzania; College of Medicine, University of Ibadan, Nigeria; University Teaching Hospital, Lusaka, Zambia

## Abstract

**Background:**

Women with anaemia have a higher risk of postpartum haemorrhage than those without anaemia. We examined the association between episiotomy and postpartum haemorrhage in women with moderate or severe anaemia.

**Methods:**

We conducted a cohort analysis using data from the World Maternal Antifibrinolytic-2 (WOMAN-2) trial. Women with moderate or severe anaemia who were giving birth vaginally were recruited from 34 hospitals in Nigeria, Pakistan, Tanzania, and Zambia. On arrival at hospital, the participants’ haemoglobin concentration was measured with a validated point-of-care haemoglobin test. Moderate anaemia was defined as a haemoglobin concentration of 70–99 g/L and severe anaemia as a haemoglobin concentration lower than 70 g/L. Episiotomy was defined as any perineal incision during delivery. Women were excluded from the WOMAN-2 trial if they were younger than 18 years and lacked consent from a guardian, had a known allergy to the trial intervention, had an indication or contraindication to the intervention, or were diagnosed with postpartum haemorrhage before the umbilical cord was clamped. The primary outcome was a clinical diagnosis of postpartum haemorrhage (estimated blood loss ≥500 mL or any blood loss causing haemodynamic instability), and the secondary outcome was calculated postpartum haemorrhage (estimated from peripartum change in haemoglobin concentration). We modelled the outcomes with multilevel logistic regression, adjusting for confounders with inverse probability of treatment weighting.

**Findings:**

Between Aug 24, 2019, and Sept 19, 2023, 4355 (28·9%) of the 15 068 women recruited to the WOMAN-2 trial had an episiotomy. In primiparous women who had non-instrumental births, the incidence of episiotomy was 81·1% in Pakistan (2703 of 3335), 63·3% (307 of 485) in Nigeria, 28·5% (69 of 242) in Zambia, and 15·1% (111 of 735) in Tanzania. Clinically diagnosed postpartum haemorrhage occurred in 1034 (6·9%) of 15 066 participants, and calculated postpartum haemorrhage occurred in 1417 (9·5%) of 14 863. After adjustment for confounders, episiotomy was associated with clinically diagnosed postpartum haemorrhage (odds ratio 1·88, 95% CI 1·33–2·66) and calculated postpartum haemorrhage (1·63, 1·14–2·34).

**Interpretation:**

Although WHO recommends against the routine use of episiotomy, many first-time mothers were given this procedure. Episiotomy might increase the risk of postpartum haemorrhage in women with moderate or severe anaemia. As anaemia is known to worsen the life-threatening complications of postpartum haemorrhage, the risks of routine episiotomy could be even greater in women with anaemia.

**Funding:**

Wellcome and the Bill & Melinda Gates Foundation.

## Introduction

Episiotomy is a surgical cut to the perineum that aims to prevent severe perineal tearing and support delivery of the baby.^1^ WHO recommends against routine or liberal use of episiotomy for non-instrumental vaginal births^2^ on the basis of evidence that routine episiotomy increases the risk of severe perineal tearing and because of a paucity of evidence that it improves other outcomes.^1,2^ Episiotomy rates vary greatly between settings and the value of this procedure remains strongly debated.^3,4^

Postpartum haemorrhage is a leading cause of maternal death worldwide.^5^ Almost 90% of maternal deaths due to haemorrhage happen in south Asia and sub-Saharan Africa.^6^ Episiotomy might increase the risk of postpartum haemorrhage^7,8^—eg, a nationwide study in Ireland estimated that episiotomy increased the risk of postpartum haemorrhage by 54%.^7^ Although some smaller cohort studies have estimated even greater effect sizes,^8^ others have found no association with postpartum haemorrhage.^9^

To our knowledge, no studies have examined the impact of episiotomy on postpartum haemorrhage in women with anaemia. Anaemia is common in pregnancy, affecting a third of pregnant women worldwide and half of pregnant women in south Asia and west and central Africa.^10^ Women with anaemia have a higher risk of postpartum haemorrhage than those without anaemia and are more likely to die if it occurs.^11–13^

We explored the use of episiotomy in a large cohort of women with moderate or severe anaemia in Nigeria, Pakistan, Tanzania, and Zambia and examined the association of this procedure with postpartum haemorrhage.

## Methods

### Study design and participants

We conducted a cohort analysis using data from the World Maternal Antifibrinolytic-2 (WOMAN-2) trial. A full description of the WOMAN-2 trial protocol has been published elsewhere.^14,15^ Women in active labour were recruited from 34 hospitals in Nigeria, Pakistan, Tanzania, and Zambia. Women with moderate or severe anaemia who were delivering vaginally were eligible to take part. In line with WHO guidance, moderate anaemia was defined as a haemoglobin concentration between 70 g/L and 99 g/L, and severe anaemia was defined as a haemoglobin concentration lower than 70 g/L.^16^ Women who were younger than 18 years and lacked consent from a guardian, had a known allergy to the intervention, had an indication or contraindication to the intervention, or were diagnosed with postpartum haemorrhage before the umbilical cord was clamped were not eligible to take part in the trial. All site teams underwent extensive training on study procedures and data collection.^15^ Data accuracy was monitored closely throughout data collection. Informed consent was obtained from the trial participants; a full description of consent procedures has been published elsewhere.^14,15^ The WOMAN-2 protocol was approved by the National Health Research Ethics Committee of Nigeria (NHREC/01/01/2007–29/09/2019); the National Bioethics Committee of Pakistan (NBC-340); the National Institute of Medical Research of Tanzania (NIMR/HQ/R.8a/Vol.IX/3767); the University of Zambia Biomedical Research Ethics Committee (REF 001–04–19); and the London School of Hygiene & Tropical Medicine’s Ethics Committee (15194).

### Procedures and outcomes

On arrival at hospital, the participants’ haemoglobin concentration was measured with the HemoCue Hb 201 + System (Hemocue; Ängelholm, Sweden)—a validated point-of-care haemoglobin test.^17,18^ Some women were weighed on admission and the weight for the other women was estimated. Episiotomy was defined as any cut made to the perineum during delivery, which was recorded from women’s medical records by research staff. We calculated the cumulative incidence of episiotomy by country, primiparity, and instrumental birth.

The primary outcome was a clinical diagnosis of postpartum haemorrhage. This diagnosis was based on an estimated blood loss of more than 500 mL or any blood loss sufficient to compromise haemodynamic stability within 24 h of delivery. Haemodynamic instability was based on signs such as tachycardia, reduced urine output, or low systolic blood pressure.

The secondary outcome was calculated postpartum haemorrhage. We calculated the volume of blood lost by multiplying the estimated total blood volume by the relative change in peripartum haemoglobin. Postpartum haemoglobin concentration was measured with the HemoCue 201 + System 24 h after delivery or at discharge, whichever happened first.^17^ We estimated total blood volume (L) by multiplying the woman’s pregnant weight in kg by 0·085 and corrected for the effect of blood transfusion on postpartum haemoglobin using a method described by Roubinian and colleagues.^19^

Women with an estimated blood loss of 1000 mL or more were defined as having calculated postpartum haemorrhage. The primary and secondary postpartum haemorrhage outcomes were recorded for all participants.

We also examined the causes of bleeding for women with a clinical diagnosis of postpartum haemorrhage. Cases were categorised according to whether tearing, uterine atony, or retained placenta contributed to the bleeding.

### Statistical analysis

We generated a directed acyclic graph to identify potential (measured and unmeasured) confounding factors (appendix 6 p 3) and accounted for confounding with a propensity score approach.^20^ We used logistic regression to estimate the probability of women having an episiotomy (ie, the propensity score) on the basis of their baseline characteristics. These characteristics included sociodemographics, pregnancy history, maternal complications, birth and baby characteristics, and country (a full list of covariates is in appendix 6 [p 2]). We used the estimated propensity scores to calculate inverse probability of treatment weights, which we integrated into the outcome modelling strategy. We assessed covariate balance before and after weighting using absolute standardised mean differences. We added interaction terms to the propensity score model until mean differences fell to lower than 0·10 for key covariates (full model specifications are in appendix 6 [p 2]). A value higher than 0·10 is a common indicator of residual covariate imbalance.

To estimate the association between episiotomy and study outcomes, we used multilevel logistic regression with inverse probability of treatment weighting and robust variance estimators, including a random effect for hospital (appendix 6 p 2).^21^ We restricted our sample to women within the region of propensity score overlap between the exposure groups.^20^ We did a complete-case analysis as the propensity score model covariates had few missing data.

As women with anaemia cannot tolerate the same amount of blood loss as healthy women (because anaemia reduces the oxygen-carrying capacity of blood), we tested whether episiotomy had a more harmful effect on postpartum haemorrhage in women with severe versus moderate anaemia. We included an interaction term between anaemia severity and episiotomy and assessed its p value with a threshold of 0·05 for significance. We also tested for interactions with primiparity and country in post-hoc analyses.

We calculated population impact fractions to estimate the share of postpartum haemorrhage cases that could have been prevented if episiotomy was only done when indicated (ie, selective episiotomy).^1,22^ We approximated the rate of selective episiotomy three ways: mean incidence of episiotomy occurring in the selective groups of randomised trials;^1,4,23,24^ lowest incidence of episiotomy occurring in the selective groups of randomised trials;^1,4,23,24^ and lowest population-level incidence of episiotomy.^25^ These population impact fractions assume a causal relationship between episiotomy and postpartum haemorrhage.^22^

We did the following sensitivity analyses. To assess the effect of residual confounding by shoulder dystocia, we conducted a quantitative bias analysis.^26^ Shoulder dystocia is an important risk factor for postpartum haemorrhage in women having vaginal births, which is often managed with episiotomy.^27,28^ We also examined the effect of excluding women with non-overlapping propensity scores by repeating the analysis on the complete sample.

We used STATA 18 and R (version 4.2.0) for our analyses.

### Role of the funding source

The funders of the study had no role in study design, data collection, data analysis, data interpretation, or writing of the report.

## Results

Between Aug 24, 2019, and Sept 19, 2023, 4355 (28·9%) of 15 068 women recruited to the WOMAN-2 trial^14^ had an episiotomy (table 1; characteristics by primiparity and country are in appendix 6 [pp 4–9]). 3842 (29·6%) of 12 989 women with moderate anaemia and 513 (24·7%) of 2079 women with severe anaemia had an episiotomy. Women who had an episiotomy were mostly first-time mothers (3384 [77·7%] of 4355) having non-instrumental births (4098 [94·1%]). Perineal suturing was done for 4349 (99·9%) of 4355 women who had an episiotomy compared with 1394 (13·0%) of 10 711 who did not have an episiotomy; severe spontaneous perineal tears occurred in 14 (0·3%) and four (<0·1%) of these women, respectively. The babies’ characteristics by episiotomy status are in appendix 6 (p 10). 5205 (34·6%) women in the study had a small vulnerable newborn (any one of preterm, low birthweight, or small-for-gestational age or a combination thereof)—1362 (31·3%) of women who had an episiotomy and 3843 (36·0%) of women who did not have an episiotomy. 1036 (6·9%) of 15 068 women overall had a stillbirth—161 (3·7%) of women who had an episiotomy and 875 (8·2%) of those who did not have an episiotomy (appendix 6 p 10). Data on clinically diagnosed postpartum haemorrhage were missing for two women, and data on calculated postpartum haemorrhage were missing for 205 women. Data on parity, birthweight, and perineal sutures were missing for two women; 34 women were missing data on small-for-gestational age, and 85 were missing data on labour duration. All other variables were complete.

**Figure 1:**
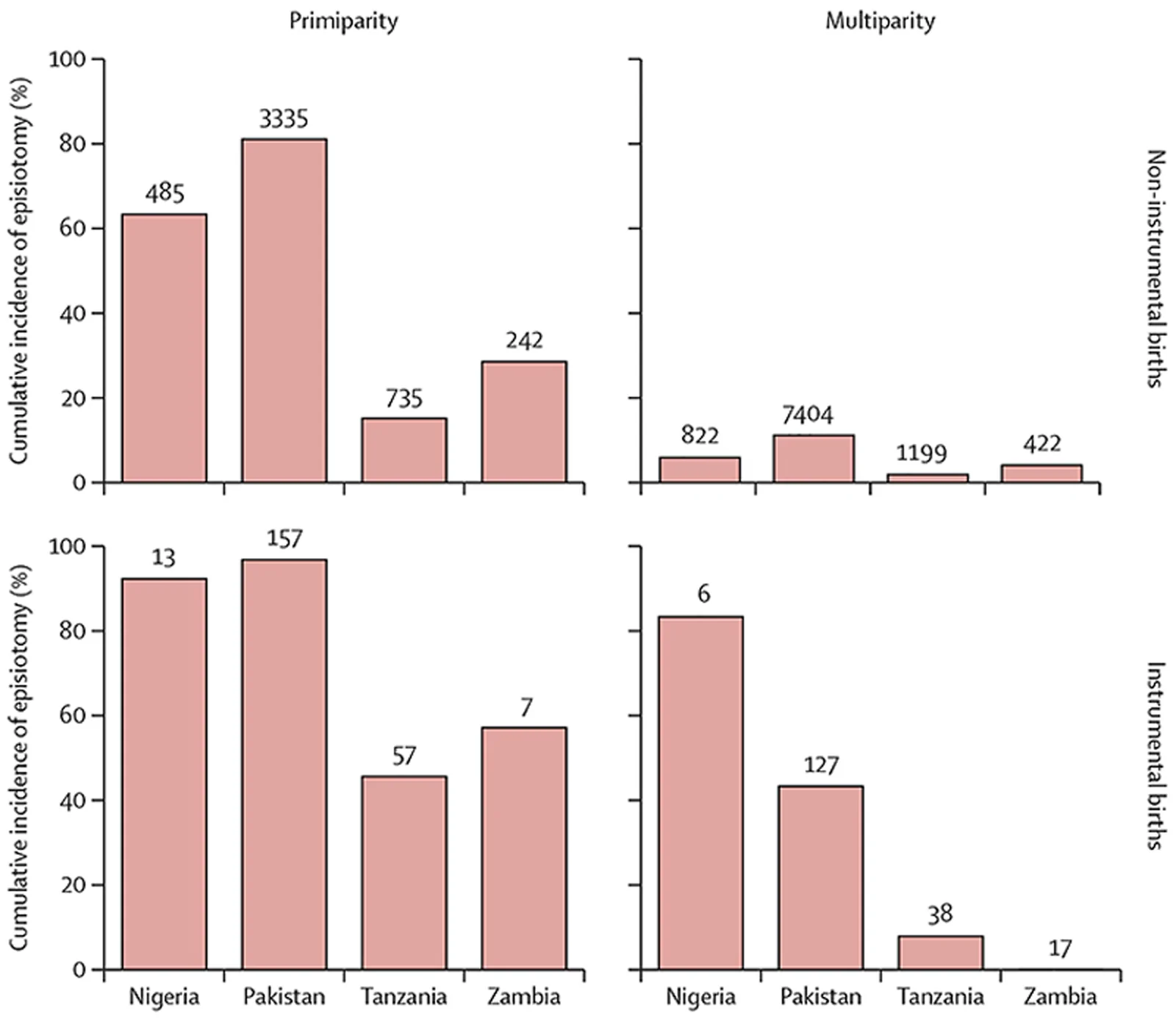
Cumulative incidence of episiotomy by country, primiparity, and instrumental birth The numbers above each column are the total numbers of births in each category.

Figure 1 shows the cumulative incidence of episiotomy by country, primiparity, and instrumental birth. In Pakistan and Nigeria, most primiparous women with non-instrumental births had an episiotomy (81·1% [2703 of 3335] of women in Pakistan and 63·3% [307 of 485] women in Nigeria). In Zambia and Tanzania, the cumulative incidences of episiotomy were 28·5% (69 of 242) and 15·1% (111 of 735), respectively (the characteristics of primiparous women by country are in appendix 6 [p 11]). Episiotomy was less common in multiparous women, ranging from 1·8% (22 of 1199) of non-instrumental births in Tanzania to 11·1% (821 of 7404) in Pakistan. Episiotomy incidence was higher in women who had instrumental versus non-instrumental births in most settings, although few women had instrumental births.

The cumulative incidence of clinically diagnosed postpartum haemorrhage was 6·9% (1034 of 15 066 participants; table 2): 8·3% (360 of 4355) in women who had an episiotomy and 6·3% (674 of 10 711) in women who did not have an episiotomy.

Tearing was a contributing cause of postpartum haemorrhage in 142 (39·4%) of 360 women who had an episiotomy and 140 (20·8%) of 674 women who did not have an episiotomy. The cumulative incidence of calculated postpartum haemorrhage was 9·5% (1417 of 14 863 participants): 9·7% (415 of 4270) in women who had an episiotomy and 9·5% (1002 of 10 593) in women who did not have an episiotomy.

**Figure 2:**
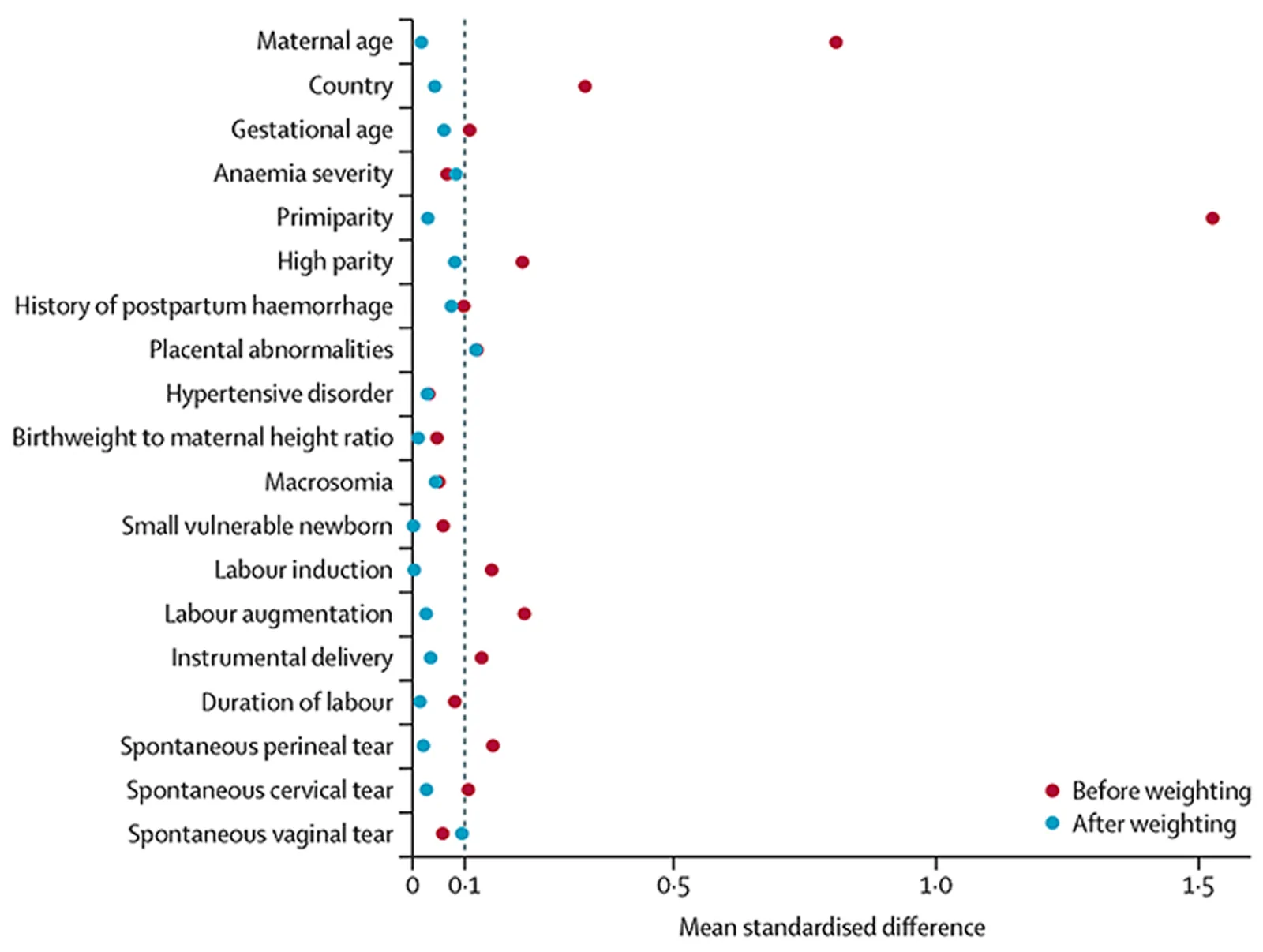
Balance of key covariates before and after propensity score weighting The vertical dashed line marks a mean standardised difference of 0·1; a value higher than this is a common indicator of residual covariate imbalance.

Inverse probability of treatment weighting reduced covariate imbalance between exposure groups (figure 2; appendix 6 p 12). After we excluded 892 women with propensity scores outside the region of overlap (participants’ characteristics are in appendix 6 [p 13]), 14 056 participants were included in the clinically diagnosed postpartum haemorrhage analyses and 13 863 were included in the calculated postpartum haemorrhage analyses. After adjusting for potential confounders, episiotomy was associated with increased risks of clinically diagnosed postpartum haemorrhage (odds ratio [OR] 1·88, 95% CI 1·33–2·66) and calculated postpartum haemorrhage (1·63, 1·14–2·34; table 3). We obtained similar results when the 892 women with propensity scores outside the region of overlap were included (appendix 6 p 14). The ORs were marginally reduced when we corrected for residual confounding by shoulder dystocia (appendix 6 p 15). Episiotomy was associated with increased odds (adjusted) of clinically diagnosed postpartum haemorrhage attributed to tearing (1·96, 1·09–3·52) and uterine atony (1·62, 1·05–2·48; appendix 6 p 16).

ORs for the association between episiotomy and clinically diagnosed postpartum haemorrhage were 1·72 for women with moderate anaemia (95% CI 1·24–2·38) and 2·20 for women with severe anaemia (0·86–5·62; p value for interaction >0·05). Covariate imbalance remained after weighting in the severe anaemia subgroup (appendix 6 pp 17–18). We found no evidence that the association between episiotomy and clinically diagnosed postpartum haemorrhage differed between primiparous and multiparous women (p value for interaction >0·05). Although we also found no evidence that the association differed by country (p value for interaction >0·05), the adjusted ORs ranged from 1·41 in Nigeria (0·79–2·55) to 3·37 (1·30–8·73) in Zambia (appendix 6 p 19).

The mean incidences of episiotomy reported for primiparous and multiparous women in the selective episiotomy groups of randomised trials^1,4,23,24^ are 38% and 26%, respectively (appendix 6 p 20). In this study, the incidence of episiotomy exceeded 38% in primiparous women in Pakistan and Nigeria (figure 1). Population impact fractions show that selective episiotomy could have prevented 22% of postpartum haemorrhage cases in Pakistan and 14% in Nigeria (appendix 6 p 20). Assuming a scenario whereby the true rates of selective episiotomy are lower than the mean incidences in the randomised trials, a greater share of cases might have been prevented.

## Discussion

Episiotomy is associated with an increased risk of postpartum haemorrhage in women with moderate or severe anaemia. Many first-time mothers in the study countries were given an episiotomy.

To our knowledge, this study is the first to examine the potential impact of episiotomy on postpartum haemorrhage in women with anaemia. We estimated the incidence of episiotomy in a large multicountry cohort of women with moderate or severe anaemia. Our primary outcome measure of clinically diagnosed postpartum haemorrhage has been validated in women with anaemia.^29^ Episiotomy was also associated with calculated postpartum haemorrhage, although the effect estimate was marginally smaller. This difference might reflect random error or differences in how postpartum bleeding was assessed. Whereas a clinical diagnosis of postpartum haemorrhage depends on maternal blood loss and its effect on the mother (haemodynamic instability), calculated postpartum haemorrhage estimates blood loss alone with a threshold of 1000 mL. We measured haemoglobin concentration using the HemoCue Hb 201 + System,^17^ which has high accuracy compared with laboratory measurements.^18^ Missing data were minimal and are unlikely to have substantially affected our results.

Our study had some limitations. Although the propensity score approach allowed us to adjust for a range of potential confounders, covariates such as labour duration could also be on the causal pathway (as shown by the directed acyclic graph). Quantitative bias analysis showed that the study findings were robust to confounding by shoulder dystocia, but bias from other unmeasured confounders remains possible. Analyses of interactions with anaemia severity, parity, and country could have been underpowered. Additionally, we were unable to assess the effect of episiotomy on important outcomes such as episiotomy-related perineal tearing and intrapartum stillbirth as these data were not collected. Although stillbirth was less common in women who had an episiotomy than in those who did not, this finding was likely confounded. Although we estimated the share of postpartum haemorrhages that could be avoided if episiotomy was done selectively, the true rate of selective episiotomy is unknown and will differ by population.

Episiotomy was associated with an almost two-fold increase in the risk of clinically diagnosed postpartum haemorrhage. A cohort study of 45 000 births in China found no association between episiotomy and postpartum haemorrhage.^9^ Our study was restricted to women with moderate or severe anaemia, which could explain these contrasting results. As anaemia reduces the oxygen-carrying capacity of blood, women with anaemia are less able to tolerate blood loss. Anaemia can also worsen bleeding as it reduces blood clotting and viscosity, increases cardiac output, and is associated with serious obstetric complications such as abruption and stillbirth.^11,14^ In a study of 300 000 births in Ireland, episiotomy was associated with a 50% higher risk of postpartum haemorrhage;^7^ smaller cohort studies have also found an increased risk.^8^ Although we cannot establish causation from observational studies, there are plausible mechanisms for the observed association between episiotomy and postpartum haemorrhage. For women with anaemia, even modest blood loss can lead to haemodynamic instability. Moreover, episiotomy can cause severe perineal tearing, which is a major risk factor for postpartum haemorrhage.^1^ The proportion of postpartum haemorrhage cases attributed to tearing (as a primary or contributing cause) in women who had an episiotomy was about twice as high as in women who did not have an episiotomy. The association between episiotomy and cause-specific postpartum haemorrhage was strongest for cases attributed to tearing. The association with postpartum haemorrhage due to uterine atony is probably due to the high proportion of all cases attributed to this cause.

81·1% of first-time mothers in Pakistan and 63·3% of first-time mothers in Nigeria were given an episiotomy. These high rates were probably unrelated to the participants’ anaemia status, as episiotomy was less common in women with severe anaemia, and high episiotomy rates have previously been documented in these settings.^3^ Assuming causation, selective episiotomy could have prevented at least 22% of postpartum haemorrhage cases in first-time mothers in Pakistan and 14% of those in Nigeria. Health-care workers often report doing routine episiotomy to prevent severe perineal tears.^30^ However, this practice is not supported by current evidence. A Cochrane review concluded that routine episiotomy increases severe perineal tearing in both primiparous and multiparous women.^1,24^ A recent trial showed that routine episiotomy might have an even worse effect on severe perineal tearing in primiparous women than in multiparous women.^24^ We found no evidence that the association between episiotomy and postpartum haemorrhage differed by parity. Future research could explore why episiotomy use varied across the study countries and examine its incidence in other settings.

Future studies should establish whether the effect of episiotomy on maternal and fetal outcomes varies by anaemia status. Although the estimated effect of episiotomy on postpartum haemorrhage was greater in women with severe versus moderate anaemia in our study, residual confounding and wide CIs make this finding difficult to interpret. The causal effect of episiotomy on postpartum haemorrhage is unclear from trials, as either postpartum haemorrhage has not been measured or its incidence has been unexpectedly low.^1,2,24^ Future trials should assess postpartum haemorrhage using a validated measure. Although we found no evidence that the effect of episiotomy differed by country, the ORs for the association between episiotomy and clinically diagnosed postpartum haemorrhage varied between study countries, possibly reflecting differences in episiotomy practice that we were unable to assess (eg, method of episiotomy and timing of incision and repair). Future studies could examine how episiotomy characteristics influence its impact on postpartum haemorrhage.

Our study showed that episiotomy might increase the risk of postpartum haemorrhage in women with moderate or severe anaemia. Postpartum haemorrhage occurs rapidly in women with anaemia, which complicates its management and limits treatment options.^14^ A survey showed that the risk of death after a postpartum haemorrhage was approximately seven times higher in women with severe anaemia than in those without severe anaemia.^12^ Another study, a multicountry survey, found that maternal death or near miss following postpartum haemorrhage was almost three times as common in women with anaemia as it was in those without anaemia.^13^ Although WHO advises against routine episiotomy (as the risks outweigh any potential benefits),^2^ in this study, most first-time mothers in Pakistan and Nigeria received an episiotomy. The risks of routine episiotomy might be even greater for women with anaemia, who are highly vulnerable to postpartum haemorrhage and its life-threatening complications.

## Supplementary Material

Appendix 1

Appendix 2

Appendix 3

Appendix 4

Appendix 5

Appendix 6

Appendix 7

Video

## Figures and Tables

**Table 1:** Participant characteristics, by episiotomy status

	Participants with episiotomy (n=4355)	Participants without episiotomy (n=10 713)	All participants (n=15 068)
Maternal age, years	24·04 (4·20)	28·48 (5·61)	27·20 (5·61)
Maternal age range, years			
<20	487 (11·18%)	408 (3·81%)	895 (5·94%)
20–29	3352 (76·97%)	5585 (52·13%)	8937 (59·31%)
30–39	504 (11·57%)	4326 (40·38%)	4830 (32·05%)
>40	12 (0·28%)	394 (3·68%)	406 (2·69%)
Country			
Nigeria	372 (8·54%)	954 (8·91%)	1326 (8·80%)
Pakistan	3731 (85·67%)	7294 (68·09%)	11 025 (73·17%)
Tanzania	162 (3·72%)	1867 (17·43%)	2029 (13·47%)
Zambia	90 (2·07%)	598 (5·58%)	688 (4·57%)
Gestational age, weeks	37·78 (2·07)	37·22 (2·87)	37·39 (2·68)
Haemoglobin at baseline, g/L	83·10 (10·89)	82·59 (12·21)	82·74 (11·84)
Anaemia			
Moderate anaemia	3842 (88·22%)	9147 (85·38%)	12 989 (86·20%)
Severe anaemia	513 (11·78%)	1566 (14·62%)	2079 (13·80%)
Parity			
One	3384 (77·70%)	1647 (15·37%)	5031 (33·40%)
Two to four	929 (21·33%)	6341 (59·19%)	7270 (48·25%)
Five to six	34 (0·78%)	1850 (17·27%)	1884 (12·50%)
Seven or more	8 (0·18%)	873 (8·15%)	881 (5·85%)
Missing	0	2 (0.02%)	2 (0.01%)
Height, m	1·58 (0·06)	1·59 (0·06)	1·59 (0·06)
BMI tertiles*			
Lowest	1591 (36·53%)	3445 (32·16%)	5036 (33·42%)
Medium	1511 (34·70%)	3520 (32·86%)	5031 (33·39%)
Highest	1253 (28·77%)	3748 (34·99%)	5001 (33·19%)
History of postpartum haemorrhage			
Yes	12 (0·28%)	140 (1·31%)	152 (1·01%)
No	4343 (99·72%)	10 573 (98·69%)	14 916 (98·99%)
History of caesarean			
Yes	217 (4·98%)	494 (4·61%)	711 (4·72%)
No	4138 (95·02%)	10 218 (95·38%)	14 356 (95·27%)
Missing	0	1 (0.01%)	1 (0.01%)
Antepartum haemorrhage			
Yes	56 (1·29%)	379 (3·54%)	435 (2·89%)
No	4299 (98·71%)	10 334 (96·46%)	14 633 (97·11%)
Placental abnormalities			
Abruption	57 (1·31%)	368 (3·44%)	425 (2·82%)
Abruption and previa	1 (0.02%)	5 (0·05%)	6 (0·04%)
Previa	2 (0·05%)	33 (0·31%)	35 (0·23%)
Accreta	0	3 (0·03%)	3 (0·02%)
None	4295 (98·62%)	10 304 (96·18%)	14 599 (96·89%)
Hypertensive disorder			
Yes	291 (6·68%)	855 (7·98%)	1146 (7·61%)
No	4064 (93·32%)	9858 (92·02%)	13 922 (92·39%)
Diabetes disorder			
Yes	28 (0·64%)	61 (0·57%)	89 (0·59%)
No	4327 (99·36%)	10 652 (99·43%)	14 979 (99·41%)
Current long-term infection			
Yes	84 (1·93%)	468 (4·37%)	552 (3·66%)
No	4271 (98·07%)	10 245 (95·63%)	14 516 (96·34%)
Polyhydramnios			
Yes	24 (0·55%)	66 (0·62%)	90 (0·60%)
No	4331 (99·45%)	10 647 (99·38%)	14 978 (99·40%)
Uterine rupture			
Yes	7 (0·16%)	10 (0·09%)	17 (0·11%)
No	4348 (99·84%)	10 703 (99·91%)	15 051 (99·89%)
Birthweight to maternal height ratio, g/m^2^	11·40 (1·99)	11·14 (2·46)	11·22 (2·34)
Birthweight, g	2847·28 (459·90)	2797·64 (590·78)	2811·99 (556·56)
Macrosomia			
Yes	52 (1·19%)	183 (1·71%)	235 (1·56%)
No	4303 (98·81%)	10 528 (98·27%)	14 831 (98·43%)
Missing	0	2 (0·02%)	2 (0·01%)
Small vulnerable newborn			
Yes	1362 (31·27%)	3843 (35·87%)	5205 (34·54%)
No	2987 (68·59%)	6844 (63·88%)	9831 (65·24%)
Missing	6 (0·14%)	26 (0·24%)	32 (0·21%)
Multiple gestation			
Yes	114 (2·62%)	469 (4·38%)	583 (3·87%)
No	4241 (97·38%)	10 244 (95·62%)	14 485 (96·13%)
Labour induction			
Not induced	3659 (84·02%)	9578 (89·41%)	13 237 (87·85%)
Induced at term	588 (13·50%)	809 (7·55%)	1397 (9·27%)
Induced preterm	108 (2·48%)	326 (3·04%)	434 (2·88%)
Labour augmentation			
Yes	1651 (37·91%)	2842 (26·53%)	4493 (29·82%)
No	2704 (62·09%)	7871 (73·47%)	10 575 (70·18%)
Instrumental birth			
Forceps	105 (2·41%)	14 (0·13%)	119 (0·79%)
Ventouse	131 (3·01%)	99 (0·92%)	230 (1·53%)
Forceps and ventouse	5 (0·11%)	0	5 (0·03%)
Other	16 (0·37%)	52 (0·49%)	68 (0·45%)
None	4098 (94·1%)	10 548 (98·5%)	14646 (97·2%)
Pharmacological pain control			
Yes	745 (17·11%)	1065 (9·94%)	1810 (12·01%)
No	3610 (82·89%)	9648 (90·06%)	13 258 (87·99%)
Duration of labour, h	13·90 (14·42)	12·48 (14·91)	12·89 (14·78)
Spontaneous perineal tear			
No tear	3963 (91·00%)	9317 (86·97%)	13 280 (88·13%)
Minor tear (first or second degree)	378 (8·68%)	1392 (12·99%)	1770 (11·75%)
Severe tear (third or fourth degree)	14 (0·32%)	4 (0·04%)	18 (0·12%)
Spontaneous cervical tear			
Yes	157 (3·61%)	177 (1·65%)	334 (2·22%)
No	4198 (96·39%)	10 536 (98·35%)	14 734 (97·78%)
Spontaneous vaginal tear			
Yes	73 (1·68%)	90 (0·84%)	163 (1·08%)
No	4282 (98·32%)	10 623 (99·16%)	14 905 (98·92%)

**Table 2:** Cumulative incidence of clinically diagnosed postpartum haemorrhage and its suspected causes, by episiotomy status

	Participants with episiotomy (N=4355)	Participants without episiotomy (N=10 711)	All participants (N=15 066)*
**Clinically diagnosed postpartum haemorrhage**			
Yes	360 (8·27%)	674 (6·29%)	1034 (6·86%)
No	3995 (91·73%)	10 037 (93·71%)	14 032 (93·14%)
**Primary cause of postpartum haemorrhage**			
Atony	224/360 (62·22%)	469/674 (69·58%)	693/1034 (67·02%)
Tears	101/360 (28·06%)	93/674 (13·80%)	194/1034 (18·76%)
Retained placental tissue	23/360 (6·39%)	77/674 (11·42%)	100/1034 (9·67%)
Placental abnormalities	1/360 (0·28%)	14/674 (2·08%)	15/1034 (1·45%)
Uterine rupture	1/360 (0·28%)	0	1/1034 (0·10%)
Unknown	4/360 (1·11%)	11/674 (1·63%)	15/1034 (1·45%)
Other	6/360 (1·67%)	10/674 (1·48%)	16/1034 (1·55%)
**Contributing cause of postpartum haemorrhage†**			
Atony	247/360 (68·61%)	505/674 (74·93%)	752/1034 (72·73%)
Tears	142/360 (39·44%)	140/674 (20·77%)	282/1034 (27·27%)
Retained placental tissue	61/360 (16·94%)	150/674 (22·26%)	211/1034 (20·41%)
Placental abnormalities	3/360 (0·83%)	17/674 (2·52%)	20/1034 (1·93%)
Uterine rupture	1/360 (0·28%)	1/674 (0·15%)	2/1034 (0·19%)
Other cause	10/360 (2·78%)	18/674 (2·67%)	28/1034 (2·71%)

Data are n (%) or n/N (%).

* Data on clinically diagnosed postpartum haemorrhage were missing for two participants.

† Contributing causes do not sum to 100%.

**Table 3:** Association of episiotomy with clinically diagnosed and calculated postpartum haemorrhage

	Participants with outcome, n/N (%)			Odds ratio (95% CI)			Intraclass correlation coefficient (95% CI)	
	Episiotomy	No episiotomy		Unadjusted	Adjusted		Unadjusted	Adjusted
Clinically diagnosed postpartum haemorrhage	330/4230 (7·80%)	605/9826 (6·16%)		1·31 (1·11–1·55)	1·88 (1·33–2·66)		8% (4–16)	24% (14–37)
Calculated postpartum haemorrhage	392/4145 (9·46%)	897/9718 (9·23%)		1·26 (1·09–1·46)	1·63 (1·14–2·34)		11% (6–18)	24% (16–34%)

Unadjusted and adjusted models include a random effect for hospital. Adjusted model also adjusts for confounders using inverse propensity score weighting (figure 2). N=14 056 for clinically diagnosed postpartum haemorrhage; N=13 863 for calculated postpartum haemorrhage.

## Data Availability

Individual de-identified WOMAN-2 trial patient data, including a data dictionary, will be made available via our data sharing portal, the Free Bank of Injury and Emergency Research Data (freebird.lshtm.ac.uk) website.

## Research in context

### Evidence before this study

Routine episiotomy increases the risk of severe perineal tearing, which is a risk factor for postpartum haemorrhage. Women with anaemia have an increased risk of postpartum haemorrhage. We searched PubMed, MedRxiv, and BioRxiv from database inception to Feb 20, 2025, with the terms “episiotomy”, “postpartum haemorrhage”, and “anaemia”, without language restrictions. We found no relevant studies.

### Added value of this study

To our knowledge, this study is the first to explore the potential impact of episiotomy on postpartum haemorrhage in women with anaemia. We estimated the incidence of episiotomy in women in Nigeria, Pakistan, Tanzania, and Zambia, which have high rates of anaemia and maternal mortality due to bleeding. In Pakistan and Nigeria, most first-time mothers were given an episiotomy. We found that episiotomy was associated with an increased risk of postpartum haemorrhage in women with moderate or severe anaemia. This finding was consistent across subjective and objective measures of postpartum haemorrhage and following multiple sensitivity analyses. 40% of postpartum haemorrhage cases were attributed to tearing in women with episiotomy.

### Implications of all the available evidence

WHO recommends against routine episiotomy because the risks outweigh any potential benefits. Despite this recommendation, many first-time mothers in our study were given an episiotomy. The risks of routine episiotomy could be even greater for women with anaemia, who experience postpartum haemorrhage faster, have fewer treatment options, and are at increased risk of its life-threatening complications, including maternal death, compared with individuals who do not have anaemia. An individual with lived experience of episiotomy and postpartum haemorrhage has shared her story (video).
