# Supplementary material for: Episiotomy and postpartum haemorrhage in women with moderate or severe anaemia: a cohort analysis of data from the WOMAN-2 trial
Source: Lancet Glob Health. Author manuscript; Available in PMC 2026 Oct 30. (PMC7619659; doi:10.1016/S2214-109X(25)00449-8)
Supplement: Supplementary file 1 [file EMS218565-supplement-Appendix_1.docx]

# Supplementary appendix 1

This translation in Swahili was submitted by the authors and we reproduce it as supplied. It has not been peer reviewed. The Lancet’s editorial processes have only been applied to the original in English, which should serve as reference for this manuscript.

Tafsiri hii katika Swahili iliwasilishwa na waandishi na tunatengeneza tena kama hutolewa. Haijapitiwa. Mchakato wa hariri wa Lancet Global Health umetumika tu kwa asili kwa Kiingereza, ambayo inapaswa kutumika kama kumbukumbu kwa muswada hii.

Supplement to: The WOMAN-2 Trial Collaborators. Episiotomy and postpartum haemorrhage in women with moderate or severe anaemia: a cohort analysis of data from the WOMAN-2 trial. Lancet Glob Health 2026; 14: e224–32.

## Abstract – Swahili

**Ikisiri**

Utangulizi

Wanawake walio na upungufu wa damu wapo katika hatari zaidi ya kutokwa na damu kupita kiasi baada wa kujifungua (Postpartum haemorrhage) kuliko wale wasiokuwa na upungufu wa damu. Tulichunguza uhusiano baina ya upasuaji wa episiotomi na na utokwaji wa damu kupita kiasi baada ya kujifungua katika wanawake walio na upungufu wa damu wa kiwango cha kati na upungufu mkubwa.

Mbinu za Utafiti Mbinu

Tuliendesha uchanganuzi wa vikundi kwa kutumia data kutokana na jaribio la World Maternal Antifibrinolytic-2 (WOMAN 2). Wanawake walio na upungufu wa damu wa kiwango cha kati na upungufu mkubwa ambao walijifungua kupitia kwenye uke walisajiliwa kutoka hospitali nchini Nigeria, Pakitani, Tanzania, na Zambia. Walipofika hospitalini, mkusanyiko wa hemoglobini ya washiriki ulipimwa kupitia kipimo cha hemoglobini kilichothibitishwa katika kituo cha utunzaji. Upungufu wa damu wa kiwango cha kati ulibainishwa kuwa mkusanyiko wa hemoglobini wa g/L 70-99 huku upungufu mkubwa wa damu ukiwa mkusanyiko wa hemoglobini ulio chini ya g/L70. Upasuaji wa episiotomi ulifafanuliwa kuwa ni ukataji wa sehemu baina ya tupu ya mbele na tupu ya nyuma wakati wa kujifungua. Jaribio la WOMAN-2 halikujumuisha wanawake walio chini ya umri wa miaka 18 na wasiokuwa na kibali kutoka kwa walezi wao, wanawake waliokuwa na mzio unaojulikana wa dawa ya jaribio, waliokuwa na dalili au waliokuwa na sababu ya kuzuia dawa ya jaribio, au waliogunduliwa kuwa wanatokwa na damu baada ya kujifungua kabla ya kitovu kufungwa. Matokeo ya msingi yalikuwa utambuzi wa kimatibabu wa utokwaji wa damu kupita kiasi baada ya kujifungua (makadirio ya upotevu wa damu wa mililita

≥500 au upotevu mwingine wa damu unaosababisha ukosefu wa uthabiti wa viwango vya damu) na matokeo ya upili yalikuwa ukadiriaji wa utokwaji wa damu kupita kiasi baada ya kujifungua (makadirio kutokana na mabadiliko ya mkusanyiko wa himoglobini katika kipindi kabla ya kujifungua). Tuliyaeleza matokeo kwa kutumia uregu wa ngazi nyingi, tukirekebisha kwa ajili ya mkanganyiko wa athari ya matibabu kwa kutumia welekeo kinyume.

Matokeo

Baina ya tarehe 24 Agosti, 2029, na tarehe 19 Septemba, 2023, wanawake 4355 (asilimia 28.9%) kati ya wanawake 15,068 waliosajiliwa katika jaribio la WOMAN-2 walifanyiwa upasuaji wa episiotomi.. Katika wanawake waliojifungua mtoto wa kwanza baada ya kupoteza watoto katika uzazi hapo awali, matukizi ya upasuaji wa episiotomi yalikuwa asilimia 81·1% nchini Pakistani (2703 kati ya 3335), asilimia 63·3% (307 kati ya 485) nchini Nigeria, asilimia 28·5% (69 kati ya 242) nchini Zambia, na asilimia 15·1% (111 kati ya 735) nchini Tanzania.

Utokwaji wa damu kupita kiasi baada ya kujifungua uligunduliwa kimatibabu kwa washiriki 1034 (asilimia 6·9%) kati ya washiriki 15 066, na utokwaji wa damu kupita kiasi baada ya kujifungua ulitokea kwa washiriki 1417 (asilimia 9·5%) kati ya 14 863. Baada ya marekebisho kwa ajili ya mkanganyiko, upasuaji wa episiotomi ulihusishwa na utambuzi wa utokwaji wa damu kupita kiasi baada ya kujifungua (uwiano wa uwezekano 1 1·88, asilimia 95% CI 1·33-2·66) na utokwaji wa damu kupita kiasi baada ya kujifungua uliohesabiwa (1·63, 1·14-2·34).

Ufafanuzi

Ingawa Shirika la Afya Duniani linapendekeza kutotumia upasuaji wa episiotomi mara kwa mara, akina mama wengi wanaojifungua kwa mara ya kwanza walifanyiwa upasuaji huu. Upasuaji wa episiotomi unaweza kuongeza hatari ya utokwaji wa damu kupita kiasi katika wanawake walio na upungufu wa damu wa kiwango cha kati na upungufu mkubwa. Kwa kuwa upungufu wa damu unahusishwa na changamoto za utokwaji wa damu kupita kiasi baada ya kujifungua unaotishia maisha, hatari za upasuaji wa episiotomi wa mara kwa mara zitakuwa nyingi zaidi katika wanawake walio na upungufu wa damu.

Ufadhili

Wellcome na Wakfu wa Bill & Melinda Gates.
