# Supplementary material for: Episiotomy and postpartum haemorrhage in women with moderate or severe anaemia: a cohort analysis of data from the WOMAN-2 trial
Source: Lancet Glob Health. Author manuscript; Available in PMC 2026 Oct 30. (PMC7619659; doi:10.1016/S2214-109X(25)00449-8)
Supplement: Supplementary file 2 [file EMS218565-supplement-Appendix_2.docx]

# Supplementary appendix 2

## This translation in Urdu was submitted by the authors and we reproduce it as supplied. It has not been peer reviewed. The Lancet’s editorial processes have only been applied to the original in English, which should serve as reference for this manuscript.

## Supplement to: The WOMAN-2 Trial Collaborators. Episiotomy and postpartum haemorrhage in women with moderate or severe anaemia: a cohort analysis of data from the WOMAN-2 trial. Lancet Glob Health 2026; 14: e224–32.

### ﺧﻼﺻہ

ﺗﻌﺎرف

ﺑﻧﺎ اﻧﯾﻣﯾﺎ )ﺧون ﮐﯽ ﮐﻣﯽ( واﻟﯽ ﺧواﺗﯾن ﮐﮯ ﻣﻘﺎﺑﻠﮯ ﻣﯾں اﻧﯾﻣﯾﺎ واﻟﯽ ﺧواﺗﯾن ﻣﯾں ﺑﻌد از وﻻدت ﺟرﯾﺎن ﺧون ﮐﺎ ﺧطره زﯾﺎده ﮨوﺗﺎ ﮨﮯ۔ ﮨم ﻧﮯ ﻣﻌﺗدل ﯾﺎ ﺷدﯾد اﻧﯾﻣﯾﺎ واﻟﯽ ﺧواﺗﯾن ﻣﯾں اﯾﭘﯾﺳﯾوﮢوﻣﯽ )ﻓرج ﺷﮕﺎﻓﯽ( اور ﺑﻌد از وﻻدت ﺟرﯾﺎن ﺧون ﮐﮯ ﻣﺎﺑﯾن ﺗﻌﻠﻖ ﮐﯽ ﺟﺎﻧﭻ ﮐﯽ۔

طرﯾﻘہ ﮐﺎر

ﮨم ﻧﮯ ورﻟڈ ﻣﯾﮢرﻧل اﯾﻧﮢﯽ ﻓﺎﺋﺑرﯾﻧوﻟﮢﮏ2- )ووﻣن(2- آزﻣﺎﺋش ﮐﮯ ڈﯾﮢﺎ ﮐﺎ اﺳﺗﻌﻣﺎل ﮐرﺗﮯ ﮨوﺋﮯ اﯾﮏ ﺟﻣﺎﻋﺗﯽ ﺗﺟزﯾہ ﮐﯾﺎ۔ اﻧدام ﻧﮩﺎﻧﯽ ﮐﮯ ذرﯾﻌﮯ ﺑﭼﮯ ﮐو ﺟﻧم دﯾﻧﮯ واﻟﯽ ﻣﻌﺗدل ﯾﺎ ﺷدﯾد اﻧﯾﻣﯾﺎ واﻟﯽ ﺧواﺗﯾن ﮐو ﻧﺎﺋﺟﯾرﯾﺎ، ﭘﺎﮐﺳﺗﺎن، ﺗﻧزاﻧﯾہ اور زﯾﻣﺑﯾﺎ ﮐﮯ 34 ﮨﺳﭘﺗﺎﻟوں ﺳﮯ ﺑﮭرﺗﯽ ﮐﯾﺎ ﮔﯾﺎ ﺗﮭﺎ۔ ﮨﺳﭘﺗﺎل ﭘﮩﻧﭼﻧﮯ ﭘر، ﺷرﮐﺎء ﮐﮯ ﮨﯾﻣوﮔﻠوﺑن ﮐﯽ ارﺗﮑﺎز ﮐو اﯾﮏ ﺗوﺛﯾﻖ ﺷده ﭘواﺋﻧٹ آف ﮐﯾﺋر ﮨﯾﻣوﮔﻠوﺑن ﮢﯾﺳٹ ﺳﮯ ﻣﺎﭘﺎ ﮔﯾﺎ۔ ﻣﻌﺗدل ﺧون ﮐﯽ ﮐﻣﯽ ﮐو ﮨﯾﻣوﮔﻠوﺑن ﮐﮯ 99-70 ﮔرام/ﻟﯾﮢر ﮐﮯ ارﺗﮑﺎز ﮐﮯ طور ﭘر اور ﺷدﯾد ﺧون ﮐﯽ ﮐﻣﯽ ﮐو ﮨﯾﻣوﮔﻠوﺑن ﮐﮯ 70 ﮔرام/ﻟﯾﮢر ﮐﮯ ارﺗﮑﺎز ﺳﮯ ﮐم ﮐﮯ طور ﭘر ﺑﯾﺎن ﮐﯾﺎ ﮔﯾﺎ ﺗﮭﺎ۔ اﯾﭘﯾﺳﯾوﮢوﻣﯽ ﮐﯽ ﺗﻌرﯾف وﻻدت ﮐﮯ دوران ﮐﺳﯽ ﺑﮭﯽ ﻋﺟﺎﻧﯽ ﭼﯾرے ﮐﮯ طور ﭘر ﮐﯽ ﮔﺋﯽ ﺗﮭﯽ۔ ﺧواﺗﯾن ﮐو ووﻣن2- آزﻣﺎﺋش ﺳﮯ ﺧﺎرج ﮐر دﯾﺎ ﮔﯾﺎ ﺗﮭﺎ اﮔر وه 18 ﺳﺎل ﺳﮯ ﮐم ﻋﻣر ﮐﯽ ﺗﮭﯾں اور ﮐﺳﯽ ﺳرﭘرﺳت ﮐﯽ رﺿﺎﻣﻧدی ﺳﮯ ﻣﺣروم ﺗﮭﯾں، ﺟﻧﮩﯾں آزﻣﺎﺋﺷﯽ ﻣداﺧﻠت ﺳﮯ ﻣﻌﻠوم اﻟرﺟﯽ ﺗﮭﯽ، ﺟﻧﮩﯾں ﻣداﺧﻠت ﮐﯽ ﮐوﺋﯽ ﻋﻼﻣت ﯾﺎ ﻣﺧﺎﻟف ﻋﻼﻣت ﺗﮭﯽ، ﯾﺎ ﺟﻧﮩﯾں ﻧﺎل ﺑﻧد ﮨوﻧﮯ ﺳﮯ ﭘﮩﻠﮯ ﺑﻌد از وﻻدت ﺟرﯾﺎن ﺧون ﮐﯽ ﺗﺷﺧﯾص ﮨوﺋﯽ ﺗﮭﯽ۔ ﺑﻧﯾﺎدی ﻧﺗﯾﺟہ ﺑﻌد از وﻻدت ﺟرﯾﺎن ﺧون ﮐﯽ طﺑﯽ ﺗﺷﺧﯾص ﺗﮭﯽ )ﺗﺧﻣﯾﻧہ ﺷده ﺧون ﮐﯽ ﮐﻣﯽ 500≤ ﻣﻠﯽ ﻟﯾﮢر ﯾﺎ ﺧون ﮐﯽ ﮐوﺋﯽ ﮐﻣﯽ ﺗﮭﯽ ﺟو ﮨﯾﻣوڈﯾﻧﺎﻣﮏ ﻋدم اﺳﺗﺣﮑﺎم ﮐﺎ ﺑﺎﻋث ﺑﻧﺗﯽ ﮨﮯ( اور ﺛﺎﻧوی ﻧﺗﯾﺟہ ﮐﺎ ﺣﺳﺎب ﺑﻌد از وﻻدت ﺟرﯾﺎن ﺧون ﺳﮯ ﻟﮕﺎﯾﺎ ﮔﯾﺎ ﺗﮭﺎ )ﺟس ﮐﺎ اﻧدازه ﮨﯾﻣوﮔﻠوﺑن ﮐﮯ ارﺗﮑﺎز ﻣﯾں ﭘﯾداﺋش ﮐﮯ آس ﭘﺎس ﮐﯽ ﻣدت ﮐﯽ ﺗﺑدﯾﻠﯽ ﺳﮯ ﻟﮕﺎﯾﺎ ﺟﺎﺗﺎ ﮨﮯ(۔ ﮨم ﻧﮯ ﮐﺛﯾر ﺳطﺣﯽ ﻻﺟﺳﮢﮏ رﯾﮕرﯾﺷن ﮐﮯ ﺳﺎﺗﮭ ﻧﺗﺎﺋﺞ ﮐو ﻣﺎڈل ﺑﻧﺎﯾﺎ، اور ﻋﻼج ﮐﮯ وزن ﮐﮯ اﻟﮢﺎ اﻣﮑﺎن ﮐﮯ ﺳﺎﺗﮭ ﮐﻧﻔﺎؤﻧڈرز ﮐو اﯾڈﺟﺳٹ ﮐﯾﺎ۔

ﻧﺗﺎﺋﺞ

24 اﮔﺳت 2019 اور 19 ﺳﺗﻣﺑر 2023 ﮐﮯ درﻣﯾﺎن ووﻣن2- ﮐﯽ آزﻣﺎﺋش ﻣﯾں ﺑﮭرﺗﯽ ﮨوﻧﮯ واﻟﯽ 15068 ﺧواﺗﯾن ﻣﯾں ﺳﮯ 4355 28.9) ﻓﯾﺻد( ﮐﯽ اﯾﭘﯾﺳﯾوﮢوﻣﯽ )ﻓرج ﺷﮕﺎﻓﯽ( ﮨوﺋﯽ۔ ﭘﮩﻠﯽ ﺑﺎر ﺑﭼہ ﺟﻧﻧﮯ واﻟﯽ ﺧواﺗﯾن ﻣﯾں ﺟن ﮐﯽ ﭘﯾداﺋش آﻻت ﮐﮯ ﺑﻐﯾر ﮨوﺋﯽ ﺗﮭﯽ، اﯾﭘﯾﺳﯾوﮢوﻣﯽ )ﻓرج ﺷﮕﺎﻓﯽ( ﮐﮯ واﻗﻌﺎت ﭘﺎﮐﺳﺗﺎن ﻣﯾں 81.1 ﻓﯾﺻد 3335) ﻣﯾں ﺳﮯ (2703، ﻧﺎﺋﯾﺟﯾرﯾﺎ ﻣﯾں 63.3 ﻓﯾﺻد 485) ﻣﯾں ﺳﮯ (307، زﯾﻣﺑﯾﺎ ﻣﯾں 28.5 ﻓﯾﺻد 242) ﻣﯾں ﺳﮯ (69 اور ﺗﻧزاﻧﯾہ ﻣﯾں 15.1 ﻓﯾﺻد 735) ﻣﯾں ﺳﮯ (111 ﺗﮭﮯ۔ طﺑﯽ طور ﭘر ﺗﺷﺧﯾص ﺷده ﺑﻌد از وﻻدت ﺟرﯾﺎن ﺧون 15066 ﺷرﮐﺎء ﻣﯾں ﺳﮯ 1034 6.9) ﻓﯾﺻد( ﻣﯾں ﮨوا، اور 14863 ﻣﯾں ﺳﮯ 1417 9.5) ﻓﯾﺻد( ﻣﯾں ﺷﻣﺎر ﺷده ﺑﻌد از وﻻدت ﺟرﯾﺎن ﺧون ﮨوا۔ ﮐﻧﻔﺎؤﻧڈرز ﮐو اﯾڈﺟﺳٹ ﮐرﻧﮯ ﮐﮯ ﺑﻌد، اﯾﭘﯾﺳﯾوﮢوﻣﯽ ﮐﺎ ﺗﻌﻠﻖ طﺑﯽ طور ﭘر ﺗﺷﺧﯾص ﺷده ﺑﻌد از وﻻدت ﺟرﯾﺎن ﺧون )اوڈز ﺗﻧﺎﺳب 1.88، 95 ﻓﯾﺻد 1.33–2.66 (CI اور ﺷﻣﺎر ﮐرده ﺑﻌد از وﻻدت ﺟرﯾﺎن ﺧون )اوڈز ﺗﻧﺎﺳب 1.63، (2.34-1.14 ﺳﮯ ﺗﮭﺎ۔

ﺗﺷرﯾﺢ

اﮔرﭼہ ﻋﺎﻟﻣﯽ اداره ﺻﺣت (WHO) اﯾﭘﯾﺳﯾوﮢوﻣﯽ ﮐﮯ ﻣﻌﻣول ﮐﮯ اﺳﺗﻌﻣﺎل ﮐﮯ ﺧﻼف ﺗﺟوﯾز ﮐرﺗﺎ ﮨﮯ، ﻟﯾﮑن ﺑﮩت ﺳﯽ ﭘﮩﻠﯽ ﺑﺎر ﺑﭼہ ﺟﻧﻧﮯ واﻟﯽ ﻣﺎؤں ﮐو ﯾہ طرﯾﻘہ ﮐﺎر دﯾﺎ ﮔﯾﺎ ﺗﮭﺎ۔ اﯾﭘﯾﺳﯾوﮢوﻣﯽ ﮐﯽ وﺟہ ﺳﮯ ﻣﻌﺗدل ﯾﺎ ﺷدﯾد ﺧون ﮐﯽ ﮐﻣﯽ واﻟﯽ ﺧواﺗﯾن ﻣﯾں ﺑﻌد از وﻻدت ﺟرﯾﺎن ﺧون ﮐﺎ ﺧطره ﺑڑھ ﺳﮑﺗﺎ ﮨﮯ۔ ﭼوﻧﮑہ اﻧﯾﻣﯾﺎ ﮐو ﺑﻌد از وﻻدت ﺟرﯾﺎن ﺧون ﮐﯽ ﺟﺎن ﻟﯾوا ﭘﯾﭼﯾدﮔﯾوں ﮐو ﺧراب ﮐرﻧﮯ ﮐﮯ ﻟﯾﮯ ﺟﺎﻧﺎ ﺟﺎﺗﺎ ﮨﮯ، اس ﻟﯾﮯ اﻧﯾﻣﯾﺎ واﻟﯽ ﺧواﺗﯾن ﻣﯾں ﻣﻌﻣول ﮐﯽ اﯾﭘﯾﺳﯾوﮢوﻣﯽ ﮐﮯ ﺧطرات اور ﺑﮭﯽ زﯾﺎده ﮨو ﺳﮑﺗﮯ ﮨﯾں۔

ﻓﻧڈﻧﮓ

وﯾﻠﮑم اور ﺑل اﯾﻧڈ ﻣﯾﻠﻧڈا ﮔﯾﮢس ﻓﺎؤﻧڈﯾﺷن۔

JL Abstract (Post-proof) Urdu – 17 December 2025
