# Supplementary material for: Episiotomy and postpartum haemorrhage in women with moderate or severe anaemia: a cohort analysis of data from the WOMAN-2 trial
Source: Lancet Glob Health. Author manuscript; Available in PMC 2026 Oct 30. (PMC7619659; doi:10.1016/S2214-109X(25)00449-8)
Supplement: Supplementary file 3 [file EMS218565-supplement-Appendix_3.docx]

# Supplementary appendix 3

## This translation in Yoruba was submitted by the authors and we reproduce it as supplied. It has not been peer reviewed. The Lancet’s editorial processes have only been applied to the original in English, which should serve as reference for this manuscript.

## Supplement to: The WOMAN-2 Trial Collaborators. Episiotomy and postpartum haemorrhage in women with moderate or severe anaemia: a cohort analysis of data from the WOMAN-2 trial. Lancet Glob Health 2026; 14: e224–32.

### Akótán

Ìṣáájú

Àwọn obìrin tí wọ́n ní àìsàn ẹ̀jẹ̀ tí kò pọ̀ tó wà nínú ewu ńlá làti ní àrùn ẹ̀jẹ̀ ṣíṣàn lẹ́yìn ìbímọ ju àwọn tí kò ní àìsàn ẹ̀jẹ̀ tí kò pọ̀ tó lọ. A ṣe àyèwò ìbáṣepọ̀ tí ó wà láàrín gígé ojú ara obìrin àti ẹ̀jẹ̀ ṣíṣàn lẹ́yìn ìbímọ nínú àwọn obìrin tí ẹ̀jẹ̀ wọn kò pọ̀ tó níwọ̀nba tàbí àwọ́n tí tiwọn peléke.

Àwọn ìlànà

A ṣe àtúpalẹ̀ àkọsílẹ̀ àwọn àyẹ̀wò tí wọ́n ti gbà sílẹ̀ láti Àjọ Àgbáyé tí ó tí ń ṣe àbòjútó Oògùn tí ó ńdá ẹ̀jẹ̀ dúró lára Ìyá (OBÌRIN- MÉJÌ). Àwọn obìrin tí ẹ̀jẹ̀ wọn kò pọ̀ tó níwọ̀nba tàbí àwọ́n tí tiwọn peléke tí wọn sì ńbímọ wọ́ọ́rọ́wọ́ mẹ́iìnlélọ́gbọ̀n ni a pè fún àyẹ̀wò láti àwọn ilé ìwòsàn ní Nigeria, Pakistan, Tanzania, àti Zambia. Ní gẹ́rẹ́ tí wọ́n dé ilé ìwòsàn, a ṣe àyẹ̀wò ìwọ̀n ìpele afẹ́fẹ́ ìmí tí ó ńlọ káàkiri inú ẹ̀jẹ̀ nípa ìtọ̀jú lẹ́sẹ̀kẹsẹ̀ pẹ̀lú àbájáde àyẹ̀wò ìwọ̀n ìpele afẹ́fẹ́ ìmí tí ó dájú. Ẹ̀jẹ̀ tí kò pọ̀ tó níwọ̀nba ni a pè ní afẹ́fẹ́ ìmí tí ó ńlọ káàkiri inú ẹ̀jẹ̀ 70–99 g/L àti èyí tí ó peléke ní afẹ́fẹ́ ìmí tí ó ńlọ káàkiri inú

ẹ̀jẹ̀ 70 g/L. Àtọwọ́dá mímú ojú ara obìnrin gbòòro fún ọmọ láti jáde ni’rọrùn lásìkòo ìbímọ ni wọ́n npè ní Gígé ojú ara obìnrin. A yọ àwọn obìrin kúrò nínú ìdánwò OBÌRIN-2 tí ọjọ́ orí wọn kò bá tíì pé ọdún méjìdínlógún àti tí a kò fún ní ààyè láti ọ̀dọ̀ olùtọ́jú wọn, tàbí tí ìdánwò náà kò bá lára mu, tí wọn ní àfihàn tàbí ìdí tí kò yẹ kí wọ́n kópa ní ètò náà, tàbí tí àyẹ̀wò fihàn pé wọn ti ní ẹ̀jẹ̀ ṣíṣàn lẹ́yìn ìbímọ kí a tó di òkùn ìwọ́. Ìdí àbájáde pàtàkì èkíní àti àbájáde èkejì ni àwọn àyẹ̀wò ilé ìwòsàn fún ẹ̀jẹ̀ ṣíṣàn lẹ́yìn ìbímọ (ìṣirò àfojúsùn pípàdánù ẹ̀jẹ̀ ≥500 mL tàbí pípàdánù ẹ̀jẹ̀ tí ó ń fa àìdúró ṣìṣàn ẹ̀jẹ̀) àti àbájáde èkejì ni àkíyèsí ẹ̀jẹ̀ ṣíṣàn lẹ́yìn ìbímọ (àfojúsùn Ìyípadà nínú ìkànsí ẹ̀jẹ̀ ní àkókò tàbí lẹ́yìn ìbímọ). A ṣe àpẹẹrẹ àbájáde pẹ̀lú ìtúpalẹ̀ onípò púpọ̀ ètò tí a yípadà, ṣíṣe àtúnṣe sí àwọn olùdásílẹ̀ pẹ̀lú àfojúsùn fún ìwọ̀n ìtọ́jú.

Àwọn àbájáde

Láàárín ọjọ́ kẹrìnlélógún òṣù kẹjọ́, ẹgbàa ọdún ó lé mọ́kàndínlógún, àti ọjọ́ mọ́kàndínlógún, ẹgbàa ọdún ó lé mẹ́tàlélógún, ẹẹ́rin ẹẹ́ta aárùń aárùń (28.9%) àwọn ẹgbẹ̀rún mẹ́ẹ̀dógún, ó lé

mẹ́jọlélọ́gọ́ọ̀rin obìrin tí a ṣètò fún àyẹ̀wò OBÌRIN- MÉJÌni a ti gé ojú ara obìnrin wọn. Nínú àwọn obìrin tí ó ti bímọ lẹ́ẹ̀kan, tí kìí ṣé nípa lílo íirinṣẹ́, ìṣẹ̀lẹ̀ gígé ibi tí ọmọ ti n jade jẹ́ ìdá mọ́kànlélọ́gọ́ọ̀rin ó lé òkan nínú ọgọ́ọ̀rún (Pakistan (ẹgbàá lé ní mẹ́tàlélẹ́ẹ̀ẹdẹ́gbẹ̀rin nínú ẹgbẹ̀ẹ́dógún ó lé ọ̀ọ́dúnrún lé márùndínlógójì), ìdá mẹ́tàlélọ́gọ́ta ó lé mẹ́ta nínú ọgọ́ọ̀rún (ọ̀ọ́dúnrún lé méje nínú ẹ̀ẹ́dẹ́gbẹ̀ta ó dín máàrúndínlógún) nínú ìdá ọgọ́ọ̀rún (Nigeria, méjìdínlọ́gbọ̀n ó lé márùún nínú ìdá ọgọ́ọ̀rún

(mọ́kàndínláàdọ́rin nínú igba lé ní ogójì lé méjì) ní ), oókàndínlọ́gbọ̀n nínú ìdá ọgọ́ọ̀rún (Zambia,), àti márùúdínlógún lè kan nínú ìdá ọgọ́ọ̀rún (mẹ́sàndínlọ́gọ́fà nínú ẹ̀ẹ́dẹ́gbẹ̀rin lé márùúndínlógóòjì) ní nínú ìdá ọgọ́ọ̀rún (Tanzania.). Àyẹ̀wò ti ilé ìwòsàn ẹ̀jẹ̀ ṣíṣàn lẹ́yìn ìbímọ àti ìṣírò àyẹ̀wò wáyé ní

ẹgbẹ̀rùn lé mẹ́rìnlélọ́gbọ̀n (ẹẹ́fà·ẹẹ́sàn án nínú ìdá ọgọ́ọ̀rún) nínú ẹgbẹ̀ẹ́dógún lé mẹ́rìndínláàádọ́rin olùkópa, méje nínú ìdá ọgọ́ọ̀rún àti ìṣírò àyẹ̀wò ẹ̀jẹ̀ ṣíṣàn lẹ́yìn ìbímọ wáyé ní ẹgbẹ̀rùn lé irínwó lé mẹ́tàdínlógún (ẹẹ́sàn án·aárùún nínú ìdá ọgọ́ọ̀rún) nínú ẹgbàá méje lé ọgọ́ọ̀ta lé mẹ́ta lè

ẹgbẹ̀rin.mẹ́wàá nínú ìdá ọgọ́ọ̀rún àwọ̀n obìrin, lẹ́sẹlẹ́sẹ. Lẹ́yìn àtúntò àtúnṣe fún alájọdásílẹ̀, gígé

ojú ara obìrin ní ó ní ìbáṣepọ̀ pẹ̀lú àyẹ̀wò ti ilé ìwòsàn ẹ̀jẹ̀ ṣíṣàn lẹ́yìn ìbímọ (ìdìpọ̀ àìṣeyọrí PPH (TÀBÍ

= oókan·méjìdínláàádọ́rin, márùn ún dínlọ́gọ́ọ̀rún nínú ìdá ọgọ́ọ̀rún CI oókan·mẹ́tàlélọ́gbọ̀n–-eéjì·mẹ́rìndínlọ́gọ́óọ̀rin) and ìṣírò àyẹ̀wò ẹ̀jẹ̀ ṣíṣàn lẹ́yìn ìbímọ (oókan·PPH (TÀBÍ =

oókan·mẹ́tàlégọ́ọ̀ta, márùn ún dínlọ́gọ́ọ̀rún nínú ìdá ọgọ́ọ̀rún CI oókan·mẹ́rìnlá–-méjì·mẹ́rinlélọ́gbọ̀n).

.

Ìtúmọ̀

Bíótilẹ̀jẹ́pé ÀJỌ̀ ÌWÒSÀN ÀGBÁYÉ kéde lòdì sí gígé ojú ara obìrin, ọ̀pọ̀lọ́pọ̀ obìnrin tí ó jẹ́ pé àkọ́kọ́ bímọ wọn ni wọ́n ṣe gígé ojú abẹ́ fún. Gígé ojú abẹ́ lè mú kí ẹ̀jẹ̀ ṣíṣàn lẹ́yìn ìbímọ léwu fún obìnrin tí ẹ̀jẹ̀ rẹ̀ kò tó níwọ̀nǹba tàbí ẹni tí tirẹ̀ peléke. Gẹ́gẹ́ bí a ti mọ̀ pé ẹ̀jẹ̀ tí kò pọ̀ tó tí lè fa gígé ẹ̀mí ènìyàn kúrú èyí tí ó jẹ́ àbáyọrísí ẹ̀jẹ̀ ṣíṣàn lẹ́yìn ìbímọ, ewu tí ó wà nínú gígé ojú ara obìrin lè gájù fún àwọn obìrintì ó ní ẹ̀jẹ̀ tí kò pọ̀ tó.

Ìnáwó

Wellcome àti Bill & Belinda Gates .
