# Supplementary material for: Episiotomy and postpartum haemorrhage in women with moderate or severe anaemia: a cohort analysis of data from the WOMAN-2 trial
Source: Lancet Glob Health. Author manuscript; Available in PMC 2026 Oct 30. (PMC7619659; doi:10.1016/S2214-109X(25)00449-8)
Supplement: Supplementary file 4 [file EMS218565-supplement-Appendix_4.docx]

# Supplementary appendix 4

This translation in Hausa was submitted by the authors and we reproduce it as supplied. It has not been peer reviewed. The Lancet’s editorial processes have only been applied to the original in English, which should serve as reference for this manuscript.

Marubuta ne suka kaddamar da wannan fassarar cikin harshen Hausa, kuma mun sake buga ta kamar yadda aka kawota. Ba a sama matsala ba wajen fassara mujallar ba. An yi amfani da tsarin edita na Lancet zuwa ainihi a cikin Turanci, wanda ya kamata ya zama abin nuni ga wannan rubutun.

Supplement to: The WOMAN-2 Trial Collaborators. Episiotomy and postpartum haemorrhage in women with moderate or severe anaemia: a cohort analysis of data from the WOMAN-2 trial. Lancet Glob Health 2026; 14: e224–32.

## Takaitaccen Bayani

Gabatarwa

Matan da ke fama da karancin jini (anaemia) na fuskantar haɗarin zubar da jini mai yawa bayan haihuwa ﬁye da waɗanda basu da karancin jini (anemia). Mun yi nazari kan alaﬁar da ke tsakanin yankin faren mace a lokacin haihuwa (episiotomy) da zubar jini bayan haihuwa a cikin matan da ke da matsakaici ko tsananin karancin jini.

Hanyoyi

Mun gudanar da binciken ﬁungiyar cutar ta amfani da bayanan daga gwajin Maganin Hana Zubar Jini ga Mata Masu Haihuwa a Duniya na Biyu (WOMAN-2). An ɗauki matan da ke da matsakaici ko tsananin karancin jini waɗanda suke haihuwa ta farji daga asibitoci 34, a Najeriya, Pakistan, Tanzania, da Zambia. An bayyana Episiotomy a matsayin duk wani yanke-yanke a a kusa da faren mace a yayin haihuwa. Sa’ad da mahalartan suka isa asibiti, an auna yawan jinin (haemoglobin) da ke jikinsu ta hanyar amfani da sahihiyar na’urar gwajin jini nan take (point-of-care test). An bayyana matsakaicin karancin jini a matsayin yawan haemoglobin da ya kai 70–99 g/L, sannan aka bayyana matsanancin karancin jini a matsayin yawan haemoglobin da bai kai 70 g/L ba. An bayyana 'episiotomy' a matsayin duk wani yankin faren mace a lokacin haihuwa.

An cire mata daga gwajin WOMAN-2 idan shekarunsu ba su kai 18 ba kuma ba su da amincewar magada, ko kuma idan an san suna da rashin jituwa da maganin gwajin, ko kuma idan akwai dalilin bayarwa ko na rashin bayarwa na maganin gare su, ko kuma idan an gano suna da matsalar zubar jini bayan haihuwa kaﬁn a yanke igiyar cibiyar jariri. Babban sakamakon da aka mayar da hankali a kai shi ne gano matsalar zubar jini bayan haihuwa ta hanyar binciken likita (kimanin jinin da aka rasa ya kai ko ya ﬁce mililita 500, ko kuma duk wani zubar jini da ya haddasa rashin daidaiton yanayin jini), yayin da sakamako na biyu shi ne lissaﬁn yawan jinin da aka rasa bayan haihuwa (wanda aka kiyasta ta hanyar canjin matakin haemoglobin kaﬁn da bayan haihuwa). Mun ﬁirﬁira sakamakon tare da amfani da wata dabara ta ﬁididdiga (multilevel logistic regression), tare da daidaita abubuwan da ke haifar da bambanci ta hanyar amfani da ma'auni (inverse probability of treatment weighting).

Sakamakon Bincike

Tsakanin 24 ga Agusta, 2019, zuwa 19 ga Satumba, 2023, mata 4355 (28.9%) daga cikin mata 15,068 da aka dauka a gwajin WOMAN-2 an yi musu yankin faren a lokacin haihuwa. A cikin mata masu haihuwa na farko waɗanda suka haihu ba tare da an yi amfani da na'urori ba, yawan yankin faren a lokacin haihuwa ya kasance kashi 81.1% a ƙasar Pakistan (mata 2703 cikin 3335), kashi 63.3% (307 cikin 485) a ƙasar Najeriya, kashi 28.5% (69 cikin 242) a ƙasar Zambiya, sannan kashi 15.1% (111 cikin 735) a ƙasar Tanzaniya. An gano matsalar zubar jini bayan haihuwa ta hanyar binciken likita a jikin mahalarta 1034 (kashi 6.9%) daga cikin 15,066, sannan lissaﬁn yawan jinin da aka rasa bayan haihuwa ya nuna hakan ya faru ga mahalarta 1417 (kashi 9.5%) daga cikin 14,863. Bayan an daidaita abubuwan da ke haifar da bambanci, yankin faren mace a lokacin haihuwa yana da alaƙa da zubar jini bayan haihuwa da likita ya gano (OR = 1.88, 95% CI 1.33-2.66) da kuma zubar jini bayan haihuwa da aka ƙididdige (OR = 1.63, 95% CI 1.14-2.34).

Fassara

Duk da cewa Hukumar Laﬁya ta Duniya (WHO) ta ba da shawarar rashin amfani da : yankin

faren mace a lokacin haihuwa a matsayin tsari na yau da kullum, an yi wa mata da yawa masu

haihuwar fari wannan yankin faren. yankin fare mace na iya ﬁara haɗarin zubar jini bayan haihuwa ga matan da suke da rashin jini mai matsakaici ko mai. Kamar yadda aka sani cewa karancin jini na ﬁara muni ga illolin masu barazaba ga rai ta zubar jini bayan haihuwa, haɗarin yin yankin faren mace (episiotomy) akai-akai zai iya zama ma ﬁ girma a cikin matan da ke da karancin jini.

Tallaﬁn Kuɗi

Wellcome and the Bill & Melinda Gates Foundation.
