# Supplementary material for: Episiotomy and postpartum haemorrhage in women with moderate or severe anaemia: a cohort analysis of data from the WOMAN-2 trial
Source: Lancet Glob Health. Author manuscript; Available in PMC 2026 Oct 30. (PMC7619659; doi:10.1016/S2214-109X(25)00449-8)
Supplement: Supplementary file 5 [file EMS218565-supplement-Appendix_5.docx]

# Supplementary appendix 5

This translation in Igbo was submitted by the authors and we reproduce it as supplied. It has not been peer reviewed. The Lancet’s editorial processes have only been applied to the original in English, which should serve as reference for this manuscript.

Ntughari edemede a n’olu Igbo bu otú ndi dere ya si dee ya. Anyi edegharia ma gbasa ya Otu ahu kwa. O dibeghi onye deghariri ya. Ótu ndezi nyocha akwukwo ndi Lanceti si dee edemede a n’ asusu bekee bu ihe anyi ka ji na-eme ihe eji ama atu.

Supplement to: The WOMAN-2 Trial Collaborators. Episiotomy and postpartum haemorrhage in women with moderate or severe anaemia: a cohort analysis of data from the WOMAN-2 trial. Lancet Glob Health 2026; 14: e224–32.

## Ṅkọwaṅkenke

Okwu mmalite

Ụmụ nwanyị ndị nwere ọbara mmịkọ (anaemia) na-enwekarị ihe ize ndụ ka ukwuu maka ọbara ọgbụgba mgbe a mụsịrị nwa karịa ndị na-enweghị ọnọdụ ọbara mmịkọ (anaemia). Anyị nyochara njikọ dị n'etiti mwasawanye a na-awasawanye ahụ nwayi iji mee ka isi nwa ya pụta nke ọma (epiziotomi) na bara ọgbụgba mgbe a mụsịrị nwa n'ime ndị inyom nwere ọnọdụ ahụike ọbara mmịkọ nke dị oke ma ọ bụ nke siri ike.

Usoro

Anyị mere nyocha otu ìgwè site n'iji datà sitere na nnwale Maka Ọgwụ Mgbochi Ọbara Ịgbaze Maka Ndị Nne Ụwa Nile Nke Abụọ (WOMAN-2). E si n'ụlọ ọgwụ 34 ndị dị na Nigeria, Pakistan, Tanzania, na Zambia debanye ụmụ nwanyi ndị nwere ọnọdụ ahụike nke ọbara mmịkọ dị oke ma ọ bụ nke siri ike bụ́ ndị ji aka ha amụ nwa. Mgbe ha bịarutere n’ụlọ ọgwụ, e ji ngwa nyocha ọbara a kwadoro nke ọma wee tụọ ọnụọgụgụ haemoglobin dị n’ahụ ndị sonyere. Akọwapụtara anaemia na-agafeghị oke dị ka mkpokọta hemoglobin nke 70-99 g/L yana anaemia siri ike dị ka mkpokọta hemoglobin dị ala karịa 70 g/L. A kọwara ịwa epiziotomi dị ka mwasawanye ọ bụla a na-awasawanye ahụ nwayi mgbe ọ na-amụ nwa. A gụpụrụ ụmụ nwanyị ndị na - erubeghị afọ 18, ndị na - enweghị nkwenye nke onye nlekọta ha, ndị a maara na ha na - enwe ahụ erughị ala n' nwale ahụ a na - eme n' nnyocha ahụ, ndị nwere ihe na - egosi ma ọ bụ ihe na - emegide ntinye aka ahụ, ma ọ bụ ndị a chọpụtara na ha na - enwe ọbara ọgbụgba mgbe a mụsịrị nwa tupu a kpachie eriri afọ ha, n' ime nwale ahụ b WOMAN - 2 Nsonaazụ ndị bụ isi na nke abụọ bụ nchọpụta ụlọ ọgwụ nke bara ọgbụgba mgbe a mụsịrị nwa (ọnụ ọgụgụ ọbara furu efu ruru ma ọ bụ karịa 500 mL (otu karama) ma ọ bụ ọbara ọ bụla furu efu nke na-akpata nsogbu nke usoro ọbara na-arụ ọrụ nke ọma) na nsonaazụ nke abụọ bụ mgbakọ ọbara ọgbụgba mgbe a mụsịrị nwa (nke a gbakọrọ site na mgbanwe nke ọnụọgụ hemoglobin n'oge a na-amụ nwa). Anyị ji usoro mgbako logistic nwere ọkwa dị iche iche gosipụta nsonaazụ ndị a, na-emezi nye ihe ndị na-emetụta ya site n'iji ntụle ọgwụgwọ na-agbanwe agbanwe.

Nchọpụta

N'agbata Aug 24, 2019, na Sept 19, 2023, 4355 (28·9%) nke ụmụnwaanyị 15 068 a họpụtara maka nnwale WOMAN-2 mere mwasawanye ahụ nwayi mgbe ọ na-amụ nwa. N'ime ụmụnwaanyị ndị mụrụ nwa na-ejighi ihe eji amụ nwa, ọnụ ọgụgụ ndị e si n'ahụ ha amịpụ nwa bụ 81.1% na Pakistan (2703 n'ime 3335), 63.3% (307 n'ime 485) na Naijiria, 28.5% (69 n'ime 242) na Zambia, na 15.1% (111 n'ime 735) na Tanzania. Ọbara ọgbụgba a chọpụtara n'ụlọ ọgwụ mere na 1034 (6· 9%) nke ndị sonyere 15, 066 na ọbara ọgbụgba a gbakọrọ ọnụ mere na 1417 (9· 5%) nke ndị sonyere 14, 863. Mgbe e emezichara ihe ndị na-emetụta, a chọpụtara na ịme mwasawanye ọ bụla a na-awasawanye ahụ nwayi mgbe ọ na-amụ nwa nwere njikọ na ọbara ọgbụgba mgbe a mụsịrị nwa, a chọpụtara site na nyocha ọgwụ (ohere nke 1·88, 95% CI 1·33 - 2·66) na ọbara ọgbụgba mgbe a mụsịrị nwa a gbakọrọ (1·63, 1·14 - 2·34)

Nkọwa

Ọ bụ ezie na Òtù Ahụ Ike Ụwa na-atụ aro ka a ghara ịdị na-a awasawanye ahụ nwayi mgbe ọ na-amụ nwa mgbe nile, a na-eme ya n'ahụ́ ọtụtụ ndị inyom mụrụ nwa mbụ ha. Mwasawanye a na-awasawanye ahụ nwayi mgbe ọ na-amụ nwa (epiziotomi) nwere ike ịbawanye, ihe ize ndụ nke ọbara ọgbụgba mgbe a mụsịrị nwa n'ime ndị inyom nwere ọnọdụ ahụike ọbara mmịkọ nke dị oke ma ọ bụ nke siri ike. N'ihi na ọnọdụ ahụike ọbara mmịkọ na-eme ka nsogbu ndị ọbara ọgbụgba

mgbe a mụsịrị nwa na-akpata dịkwuo njọ, ịme epiziotomi mgbe niile nwere ike ịkpata nnukwu ihe ize ndụ nye ụmụ nwanyị ndị nwere ya.

Nkwado ego

Wellcome and the Bill & Melinda Gates Foundation.
