# Supplementary material for: Episiotomy and postpartum haemorrhage in women with moderate or severe anaemia: a cohort analysis of data from the WOMAN-2 trial
Source: Lancet Glob Health. Author manuscript; Available in PMC 2026 Oct 30. (PMC7619659; doi:10.1016/S2214-109X(25)00449-8)
Supplement: Supplementary file 6 [file EMS218565-supplement-Appendix_6.docx]

Supplementary appendix 6

This appendix formed part of the original submission and has been peer reviewed. We post it as supplied by the authors.

Supplement to: The WOMAN-2 Trial Collaborators. Episiotomy and postpartum haemorrhage in women with moderate or severe anaemia: a cohort analysis of data from the WOMAN-2 trial. Lancet Glob Health 2026; 14: e224–32.

1 **Supplementary materials**
2 **Table 1: Description of covariates included in the propensity score model**	**2**
3 **Full specification of the propensity score model**	**2**
4 **Figure 1: Directed acyclic graph (DAG) showing the potential causal pathways between episiotomy and**
5 **postpartum haemorrhage in women having vaginal births.**	**3**
6 **Table 2: Participant characteristics, by episiotomy status (primiparous women)**	**4**
7 **Table 3: Participant characteristics, by episiotomy status (multiparous women)**	**5**
8 **Table 4: Participant characteristics, by episiotomy status (Nigeria)**	**6**
9 **Table 5: Participant characteristics, by episiotomy status (Pakistan)**	**7**
10 **Table 6: Participant characteristics, by episiotomy status (Tanzania)**	**8**
11 **Table 7: Participant characteristics, by episiotomy status (Zambia)**	**9**
12 **Table 8: Baby characteristics, by episiotomy status**	**10**
13 **Table 9: Characteristics of primiparous women, by country**	**11**
14 **Table 10: Absolute mean standardised difference of potential confounders between episiotomy groups,**
15 **before and after inverse probability of treatment weighting.**	**12**
16 **Table 11: Characteristics of women with propensity scores outside the region of overlap**	**13**
17 **Table 12: The association between episiotomy and clinically diagnosed and calculated postpartum**
18 **haemorrhage, including women with propensity scores outside the region of overlap**	**14**
19 **Table 13: Quantitative bias analysis examining the impact of a binary unmeasured confounder**
20 **representing shoulder dystocia on the clinically diagnosed postpartum haemorrhage odds ratio**	**15**
21 **Table 14: Quantitative bias analysis examining the impact of a binary unmeasured confounder**
22 **representing shoulder dystocia on the calculated postpartum haemorrhage odds ratio**	**15**
23 **Table 15: The association between episiotomy and cause-specific clinically diagnosed postpartum**
24 **haemorrhage (unadjusted and adjusted)**	**16**
25 **Table 16: Absolute mean standardised difference of potential confounders between episiotomy groups,**
26 **before and after inverse probability of treatment weighting (moderate anaemia).**	**17**
27 **Table 17: Absolute mean standardised difference of potential confounders between episiotomy groups,**
28 **before and after inverse probability of treatment weighting (severe anaemia).**	**18**
29 **Table 18: The association between episiotomy and clinically diagnosed postpartum haemorrhage, by**
30 **country (unadjusted and adjusted)**	**19**
31 **Table 19: Share of clinically diagnosed postpartum haemorrhage cases that could be prevented with**
32 **selective episiotomy in primiparous women having non-instrumental births (PIF)**	**20**
33 **Table 20: Share of clinically diagnosed postpartum haemorrhage cases that could be prevented with**
34 **selective episiotomy in multiparous women having non-instrumental births (PIF)**	**20**
35 **Reference list**	**21**

36

| **Covariate** | **Definition** | **Data source** |
| --- | --- | --- |
| Country | Country of delivery (Nigeria, Pakistan, Tanzania, Zambia) | Trial records |
| Anaemia severity | Moderate (70-99 g/L) or severe anaemia (<70 g/L) on admission. | Measured by research team |
| Maternal age | Maternal age at delivery (years) | Self-reported by mother or documented from medical records |
| Gestational age | Gestational age at delivery (weeks) |  |
| Primiparity | First pregnancy carried to viable gestational age |  |
| High parity | 7+ pregnancies carried to viable gestational age, inclusive of current |  |
| History of postpartum haemorrhage | Postpartum haemorrhage in previous birth |  |
| Placental abnormalities | Placental abruption, previa, accreta, increta, and/or percreta |  |
| Hypertensive disorder | Pre-existing hypertension, gestational hypertension, eclampsia, or pre-eclampsia |  |
| Labour induction | Receipt of any method of labour induction (mechanical and/or pharmacological) |  |
| Labour augmentation | Receipt of any method of labour augmentation |  |
| Instrumental delivery | Forceps, ventouse, or other instrument to support delivery |  |
| Pharmacological pain control | Receipt of any method of pharmacological pain control during labour |  |
| Perineal tear due to the baby (‘spontaneous’) | Any cut, fracture, or other injury sustained to the perineum by the baby during labour or delivery. This includes first, second-,  third-, and fourth-degree tears. |  |
| Spontaneous cervical tear | Any cut, fracture, or other injury sustained to the cervix by the baby during labour or delivery |  |
| Spontaneous vaginal tear | Any cut, fracture, or other injury sustained to the vagina by the baby during labour or delivery |  |
| Total duration of labour | Time between onset of painful contractions and delivery (hours) | Onset time self-reported by mother. Delivery time measured  by research team |
| Ratio of birthweight to maternal height | Fetal birth weight (g) divided by maternal height (m^2^)^(1)^ | Measured by research team |
| Macrosomia | Fetal birth weight 4,000 g + |  |
| Small vulnerable newborn | Any preterm (<37 weeks) and/or small-for-gestational-age and/or low birthweight (<2,500 g) baby^(2)^ |  |

38

39 **Full specification of the propensity score model**
40 logit P(Episiotomy = 1) = β₀ + β_PerinealTear + β_Anaemia + β_Primiparity + β_HighParity + β_CMI + β_Age

41	+ β_GestAge + β_Macrosomia + β_DelTime + β_Induction + β_Augmentation + β_Instrumental + β_

42 PainControl + β_Country + β_CervicalTear + β_VaginalTear + β_PrevPPH + β_Placenta + β_HTN + β_SVN +
43 β_(PerinealTear × Anaemia) + β_(Primiparity × Country) + β_(HighParity × CMI) + β_(CMI × Anaemia) +
44 β_(Macrosomia × GestAge) + β_(DelTime × Anaemia) + β_(Induction × Anaemia) + β_(Augmentation ×
45 Anaemia) + β_(Instrumental × Anaemia) + β_(PainControl × Anaemia) + β_(Country × Anaemia) +
46 β_(CervicalTear × Anaemia) + β_(VaginalTear × Anaemia) + β_(PerinealTear × Age × GestAge) + β_(Placenta
47 × GestAge × Age × Anaemia)

48

49 Derived inverse probability of treatment weights were incorporated in a random-effects logistic regression
50 model where the treatment variable ‘episiotomy’ was the only dependent variable and hospital was included as a
51 single random effect.

53	postpartum haemorrhage in women having vaginal births.

54

55 SGA; small-for-gestational-age, LBW; low birth weight. Unmeasured factors are presented in ovals.
56 Measured factors are presented in rectangles. Maternal complications include antenatal anaemia,
57 placental abnormalities, hypertensive disorders, and history of postpartum haemorrhage. To support
58 interpretation, we have not shown all pathways between the risk-factors for episiotomy. Graph created
59 using DAGitty.^(3)^

|  | **Episiotomy** | | | |
| --- | --- | --- | --- | --- |
|  | **Yes** | **No** | **Total** | **Total N** |
|  | N=3,384 | N=1,647 | N=5,031 |  |
| Episiotomy | 3,384 (100.00%) | 0 (0.00%) | 3,384 (67.26%) | 5,031 |
| Maternal age (years) |  |  |  | 5,031 |
| Mean (SD) | 23.41 (3.84) | 23.16 (4.26) | 23.33 (3.99) |  |
| Country |  |  |  | 5,031 |
| Nigeria | 319 (9.43%) | 179 (10.87%) | 498 (9.90%) |  |
| Pakistan | 2,855 (84.37%) | 637 (38.68%) | 3,492 (69.41%) |  |
| Tanzania | 137 (4.05%) | 655 (39.77%) | 792 (15.74%) |  |
| Zambia | 73 (2.16%) | 176 (10.69%) | 249 (4.95%) |  |
| Gestational age (weeks) |  |  |  | 5,031 |
| Mean (SD) | 37.75 (2.11) | 36.55 (3.59) | 37.35 (2.74) |  |
| Anaemia severity at baseline |  |  |  | 5,031 |
| Moderate | 2,991 (88.39%) | 1,476 (89.62%) | 4,467 (88.79%) |  |
| Severe | 393 (11.61%) | 171 (10.38%) | 564 (11.21%) |  |
| History of PPH | 0 (0%) | 0 (0%) | 0 (0%) | 5,031 |
| Placental abnormalities | 41 (1.21%) | 52 (3.16%) | 93 (1.85%) | 5,031 |
| Hypertensive disorder | 233 (6.89%) | 153 (9.29%) | 386 (7.67%) | 5,031 |
| Birthweight to maternal height ratio (g/m^2^) |  |  |  |  |
| Mean (SD) | 11.26 (1.94) | 10.41 (2.79) | 10.98 (2.29) | 5,031 |
| Macrosomia | 21 (0.62%) | 12 (0.73%) | 33 (0.66%) | 5,031 |
| Small vulnerable newborn | 1,127 (33.35%) | 798 (48.57%) | 1,925 (38.33%) | 5,022 |
| Labour induction | 548 (16.19%) | 165 (10.02%) | 713 (14.17%) | 5,031 |
| Labour augmentation | 1,367 (40.40%) | 501 (30.42%) | 1,868 (37.13%) | 5,031 |
| Instrumental delivery | 194 (5.73%) | 40 (2.43%) | 234 (4.65%) | 5,031 |
| Duration of labour (hours) |  |  |  |  |
| Mean (SD) | 13.97 (13.87) | 15.23 (18.16) | 14.38 (15.41) | 5,003 |
| Spontaneous perineal tear |  |  |  | 5,031 |
| No tear | 3,081 (91.05%) | 1,088 (66.06%) | 4,169 (82.87%) |  |
| Minor tear (1st/2nd degree) | 292 (8.63%) | 556 (33.76%) | 848 (16.86%) |  |
| Severe tear (3rd/4th degree) | 11 (0.33%) | 3 (0.18%) | 14 (0.28%) |  |
| Spontaneous cervical tear | 133 (3.93%) | 30 (1.82%) | 163 (3.24%) | 5,031 |
| Spontaneous vaginal tear | 62 (1.83%) | 37 (2.25%) | 99 (1.97%) | 5,031 |
| Data are n (%) or mean (SD). SD – standard deviation. PPH – postpartum haemorrhage. | | | | |

61

|  | **Episiotomy** | | | |
| --- | --- | --- | --- | --- |
|  | **Yes** | **No** | **Total** | **Total N** |
|  | N=971 | N=9,064 | N=10,035 |  |
| Episiotomy | 971 (100.00%) | 0 (0.00%) | 971 (9.68%) | 10,035 |
| Maternal age (years) |  |  |  |  |
| Mean (SD) | 26.24 (4.62) | 29.45 (5.27) | 29.14 (5.30) | 10,035 |
| Country |  |  |  | 10,035 |
| Nigeria | 53 (5.46%) | 775 (8.55%) | 828 (8.25%) |  |
| Pakistan | 876 (90.22%) | 6,655 (73.42%) | 7,531 (75.05%) |  |
| Tanzania | 25 (2.57%) | 1,212 (13.37%) | 1,237 (12.33%) |  |
| Zambia | 17 (1.75%) | 422 (4.66%) | 439 (4.37%) |  |
| Gestational age (weeks) |  |  |  |  |
| Mean (SD) | 37.91 (1.93) | 37.35 (2.70) | 37.40 (2.64) | 10,035 |
| Anaemia severity at baseline |  |  |  | 10,035 |
| Moderate | 851 (87.64%) | 7,671 (84.63%) | 8,522 (84.92%) |  |
| Severe | 120 (12.36%) | 1,393 (15.37%) | 1,513 (15.08%) |  |
| History of PPH | 12 (1.24%) | 140 (1.54%) | 152 (1.51%) | 10,035 |
| Placental abnormalities | 19 (1.96%) | 356 (3.93%) | 375 (3.74%) | 10,035 |
| Hypertensive disorder | 58 (5.97%) | 702 (7.74%) | 760 (7.57%) | 10,035 |
| Birthweight to maternal height ratio (g/m^2^) |  |  |  |  |
| Mean (SD) | 11.91 (2.06) | 11.27 (2.37) | 11.33 (2.35) | 10,034 |
| Macrosomia | 31 (3.19%) | 171 (1.89%) | 202 (2.01%) | 10,034 |
| Small vulnerable newborn | 235 (24.23%) | 3,044 (33.66%) | 3,279 (32.75%) | 10,013 |
| Labour induction | 148 (15.24%) | 969 (10.69%) | 1,117 (11.13%) | 10,035 |
| Labour augmentation | 284 (29.25%) | 2,340 (25.82%) | 2,624 (26.15%) | 10,035 |
| Instrumental delivery | 63 (6.49%) | 125 (1.38%) | 188 (1.87%) | 10,035 |
| Duration of labour (hours) |  |  |  |  |
| Mean (SD) | 13.67 (16.19) | 11.99 (14.19) | 12.15 (14.40) | 9,978 |
| Spontaneous perineal tear |  |  |  | 10,035 |
| No tear | 882 (90.83%) | 8,227 (90.77%) | 9,109 (90.77%) |  |
| Minor tear (1st/2nd degree) | 86 (8.86%) | 836 (9.22%) | 922 (9.19%) |  |
| Severe tear (3rd/4th degree) | 3 (0.31%) | 1 (0.01%) | 4 (0.04%) |  |
| Spontaneous cervical tear | 24 (2.47%) | 147 (1.62%) | 171 (1.70%) | 10,035 |
| Spontaneous vaginal tear | 11 (1.13%) | 53 (0.58%) | 64 (0.64%) | 10,035 |
| Data are n (%) or mean (SD). SD – standard deviation. PPH – postpartum haemorrhage. | | | | |

63

|  | **Episiotomy** | | | |
| --- | --- | --- | --- | --- |
|  | **Yes** | **No** | **Total** | **Total N** |
|  | N=372 | N=954 | N=1,326 | 1,326 |
| Episiotomy | 372 (100.00%) | 0 (0.00%) | 372 (28.05%) | 1,326 |
| Maternal age (years) |  |  |  |  |
| Mean (SD) | 24.47 (4.48) | 29.73 (5.56) | 28.25 (5.78) | 1,326 |
| Gestational age (weeks) |  |  |  |  |
| Mean (SD) | 38.31 (2.01) | 37.91 (2.30) | 38.02 (2.23) | 1,326 |
| Anaemia severity at baseline |  |  |  | 1,326 |
| Moderate | 355 (95.43%) | 913 (95.70%) | 1,268 (95.63%) |  |
| Severe | 17 (4.57%) | 41 (4.30%) | 58 (4.37%) |  |
| Parity |  |  |  | 1,326 |
| 1 | 319 (85.75%) | 179 (18.76%) | 498 (37.56%) |  |
| 2-4 | 51 (13.71%) | 654 (68.55%) | 705 (53.17%) |  |
| 5-6 | 1 (0.27%) | 99 (10.38%) | 100 (7.54%) |  |
| 7+ | 1 (0.27%) | 22 (2.31%) | 23 (1.73%) |  |
| History of PPH | 3 (0.81%) | 21 (2.20%) | 24 (1.81%) | 1,326 |
| Placental abnormalities | 1 (0.27%) | 14 (1.47%) | 15 (1.13%) | 1,326 |
| Hypertensive disorder | 16 (4.30%) | 42 (4.40%) | 58 (4.37%) | 1,326 |
| Birthweight to maternal height ratio (g/m^2^) |  |  |  |  |
| Mean (SD) | 11.85 (2.03) | 11.79 (2.24) | 11.81 (2.19) | 1,325 |
| Macrosomia | 10 (2.69%) | 33 (3.46%) | 43 (3.25%) | 1,325 |
| Small vulnerable newborn | 108 (29.11%) | 273 (28.68%) | 381 (28.80%) | 1,323 |
| Labour induction | 48 (12.90%) | 100 (10.48%) | 148 (11.16%) | 1,326 |
| Labour augmentation | 211 (56.72%) | 325 (34.07%) | 536 (40.42%) | 1,326 |
| Instrumental delivery | 17 (4.57%) | 2 (0.21%) | 19 (1.43%) | 1,326 |
| Duration of labour (hours) |  |  |  |  |
| Mean (SD) | 14.24 (10.31) | 13.02 (13.32) | 13.36 (12.56) | 1,325 |
| Spontaneous perineal tear |  |  |  | 1,326 |
| No tear | 265 (71.24%) | 764 (80.08%) | 1,029 (77.60%) |  |
| Minor tear (1st/2nd degree) | 101 (27.15%) | 189 (19.81%) | 290 (21.87%) |  |
| Severe tear (3rd/4th degree) | 6 (1.61%) | 1 (0.10%) | 7 (0.53%) |  |
| Spontaneous cervical tear | 31 (8.33%) | 32 (3.35%) | 63 (4.75%) | 1,326 |
| Spontaneous vaginal tear | 20 (5.38%) | 23 (2.41%) | 43 (3.24%) | 1,326 |
| Data are n (%) or mean (SD). SD – standard deviation. PPH – postpartum haemorrhage. | | | | |

65

|  | **Episiotomy** | | | |
| --- | --- | --- | --- | --- |
|  | **Yes** | **No** | **Total** | **Total N** |
|  | **N=3,731** | **N=7,294** | **N=11,025** |  |
| Episiotomy | 3,731 (100.00%) | 0 (0.00%) | 3,731 (33.84%) | 11,025 |
| Maternal age (years) |  |  |  |  |
| Mean (SD) | 24.11 (4.13) | 28.78 (5.26) | 27.20 (5.39) | 11,025 |
| Gestational age (weeks) |  |  |  |  |
| Mean (SD) | 37.75 (2.01) | 37.13 (2.76) | 37.34 (2.55) | 11,025 |
| Anaemia severity at baseline |  |  |  | 11,025 |
| Moderate | 3,253 (87.19%) | 5,941 (81.45%) | 9,194 (83.39%) |  |
| Severe | 478 (12.81%) | 1,353 (18.55%) | 1,831 (16.61%) |  |
| Parity |  |  |  | 11,023 |
| 1 | 2,855 (76.52%) | 637 (8.74%) | 3,492 (31.68%) |  |
| 2-4 | 841 (22.54%) | 4,336 (59.46%) | 5,177 (46.97%) |  |
| 5-6 | 28 (0.75%) | 1,515 (20.78%) | 1,543 (14.00%) |  |
| 7+ | 7 (0.19%) | 804 (11.03%) | 811 (7.36%) |  |
| History of PPH | 9 (0.24%) | 89 (1.22%) | 98 (0.89%) | 11,025 |
| Placental abnormalities | 54 (1.45%) | 326 (4.47%) | 380 (3.45%) | 11,025 |
| Hypertensive disorder | 247 (6.62%) | 564 (7.73%) | 811 (7.36%) | 11,025 |
| Birthweight to maternal height ratio (g/m^2^) |  |  |  |  |
| Mean (SD) | 11.32 (1.93) | 10.95 (2.31) | 11.08 (2.19) | 11,024 |
| Macrosomia | 37 (0.99%) | 92 (1.26%) | 129 (1.17%) | 11,024 |
| Small vulnerable newborn | 1,162 (31.15%) | 2,577 (35.35%) | 3,739 (33.93%) | 11,019 |
| Labour induction | 629 (16.86%) | 889 (12.19%) | 1,518 (13.77%) | 11,025 |
| Labour augmentation | 1,342 (35.97%) | 1,902 (26.08%) | 3,244 (29.42%) | 11,025 |
| Instrumental delivery | 207 (5.55%) | 77 (1.06%) | 284 (2.58%) | 11,025 |
| Duration of labour (hours) |  |  |  |  |
| Mean (SD) | 13.62 (14.37) | 10.68 (12.56) | 11.67 (13.27) | 10,986 |
| Spontaneous perineal tear |  |  |  | 11,025 |
| No tear | 3,540 (94.88%) | 6,974 (95.61%) | 10,514 (95.37%) |  |
| Minor tear (1st/2nd degree) | 184 (4.93%) | 320 (4.39%) | 504 (4.57%) |  |
| Severe tear (3rd/4th degree) | 7 (0.19%) | 0 (0.00%) | 7 (0.06%) |  |
| Spontaneous cervical tear | 112 (3.00%) | 107 (1.47%) | 219 (1.99%) | 11,025 |
| Spontaneous vaginal tear | 48 (1.29%) | 20 (0.27%) | 68 (0.62%) | 11,025 |
| Data are n (%) or mean (SD). SD – standard deviation. PPH – postpartum haemorrhage. | | | | |

67

|  | **Episiotomy** | | | |
| --- | --- | --- | --- | --- |
|  | **Yes** | **No** | **Total** | **Total N** |
|  | N=162 | N=1,867 | N=2,029 | 2,029 |
| Episiotomy | 162 (100.00%) | 0 (0.00%) | 162 (7.98%) | 2,029 |
| Maternal age (years) |  |  |  |  |
| Mean (SD) | 22.85 (4.32) | 27.03 (6.22) | 26.70 (6.20) | 2,029 |
| Gestational age (weeks) |  |  |  |  |
| Mean (SD) | 38.05 (2.36) | 37.60 (2.97) | 37.64 (2.93) | 2,029 |
| Anaemia severity at baseline |  |  |  | 2,029 |
| Moderate | 152 (93.83%) | 1,747 (93.57%) | 1,899 (93.59%) |  |
| Severe | 10 (6.17%) | 120 (6.43%) | 130 (6.41%) |  |
| Parity |  |  |  | 2,029 |
| 1 | 137 (84.57%) | 655 (35.08%) | 792 (39.03%) |  |
| 2-4 | 22 (13.58%) | 1,028 (55.06%) | 1,050 (51.75%) |  |
| 5-6 | 3 (1.85%) | 153 (8.19%) | 156 (7.69%) |  |
| 7+ | 0 (0.00%) | 31 (1.66%) | 31 (1.53%) |  |
| History of PPH | 0 (0.00%) | 22 (1.18%) | 22 (1.08%) | 2,029 |
| Placental abnormalities | 1 (0.62%) | 56 (3.00%) | 57 (2.81%) | 2,029 |
| Hypertensive disorder | 19 (11.73%) | 187 (10.02%) | 206 (10.15%) | 2,029 |
| Birthweight to maternal height ratio (g/m^2^) |  |  |  |  |
| Mean (SD) | 12.18 (2.40) | 11.71 (2.70) | 11.75 (2.68) | 2,029 |
| Macrosomia | 5 (3.09%) | 53 (2.84%) | 58 (2.86%) | 2,029 |
| Small vulnerable newborn | 48 (30.00%) | 663 (35.76%) | 711 (35.30%) | 2,014 |
| Labour induction | 9 (5.56%) | 89 (4.77%) | 98 (4.83%) | 2,029 |
| Labour augmentation | 72 (44.44%) | 500 (26.78%) | 572 (28.19%) | 2,029 |
| Instrumental delivery | 29 (17.90%) | 66 (3.54%) | 95 (4.68%) | 2,029 |
| Duration of labour (hours) |  |  |  |  |
| Mean (SD) | 18.46 (17.85) | 17.22 (18.79) | 17.32 (18.72) | 2,007 |
| Spontaneous perineal tear |  |  |  | 2,029 |
| No tear | 118 (72.84%) | 1,176 (62.99%) | 1,294 (63.78%) |  |
| Minor tear (1st/2nd degree) | 44 (27.16%) | 688 (36.85%) | 732 (36.08%) |  |
| Severe tear (3rd/4th degree) | 0 (0.00%) | 3 (0.16%) | 3 (0.15%) |  |
| Spontaneous cervical tear | 8 (4.94%) | 30 (1.61%) | 38 (1.87%) | 2,029 |
| Spontaneous vaginal tear | 2 (1.23%) | 11 (0.59%) | 13 (0.64%) | 2,029 |
| Data are n (%) or mean (SD). SD – standard deviation. PPH – postpartum haemorrhage. | | | | |

69

|  | **Episiotomy** | | | |
| --- | --- | --- | --- | --- |
|  | **Yes** | **No** | **Total** | **Total N** |
|  | N=90 | N=598 | N=688 | 688 |
| Episiotomy | 90 (100.00%) | 0 (0.00%) | 90 (13.08%) | 688 |
| Maternal age (years) |  |  |  |  |
| Mean (SD) | 21.68 (4.41) | 27.34 (6.64) | 26.60 (6.67) | 688 |
| Gestational age (weeks) |  |  |  |  |
| Mean (SD) | 36.62 (3.34) | 36.05 (4.03) | 36.13 (3.95) | 688 |
| Anaemia severity at baseline |  |  |  | 688 |
| Moderate | 82 (91.11%) | 546 (91.30%) | 628 (91.28%) |  |
| Severe | 8 (8.89%) | 52 (8.70%) | 60 (8.72%) |  |
| Parity |  |  |  | 688 |
| 1 | 73 (81.11%) | 176 (29.43%) | 249 (36.19%) |  |
| 2-4 | 15 (16.67%) | 323 (54.01%) | 338 (49.13%) |  |
| 5-6 | 2 (2.22%) | 83 (13.88%) | 85 (12.35%) |  |
| 7+ | 0 (0.00%) | 16 (2.68%) | 16 (2.33%) |  |
| History of PPH | 0 (0.00%) | 8 (1.34%) | 8 (1.16%) | 688 |
| Placental abnormalities | 4 (4.44%) | 13 (2.17%) | 17 (2.47%) | 688 |
| Hypertensive disorder | 9 (10.00%) | 62 (10.37%) | 71 (10.32%) | 688 |
| Birthweight to maternal height ratio (g/m^2^) |  |  |  |  |
| Mean (SD) | 11.48 (2.62) | 10.61 (3.21) | 10.72 (3.15) | 688 |
| Macrosomia | 0 (0.00%) | 5 (0.84%) | 5 (0.73%) | 688 |
| Small vulnerable newborn | 44 (50.00%) | 330 (55.74%) | 374 (55.00%) | 680 |
| Labour induction | 10 (11.11%) | 57 (9.53%) | 67 (9.74%) | 688 |
| Labour augmentation | 26 (28.89%) | 115 (19.23%) | 141 (20.49%) | 688 |
| Instrumental delivery | 4 (4.44%) | 20 (3.34%) | 24 (3.49%) | 688 |
| Duration of labour (hours) |  |  |  |  |
| Mean (SD) | 16.10 (21.29) | 19.14 (23.14) | 18.73 (22.91) | 665 |
| Spontaneous perineal tear |  |  |  | 668 |
| No tear | 40 (44.44%) | 403 (67.39%) | 443 (64.39%) |  |
| Minor tear (1st/2nd degree) | 49 (54.44%) | 195 (32.61%) | 244 (35.47%) |  |
| Severe tear (3rd/4th degree) | 1 (1.11%) | 0 (0.00%) | 1 (0.15%) |  |
| Spontaneous cervical tear | 6 (6.67%) | 8 (1.34%) | 14 (2.03%) | 688 |
| Spontaneous vaginal tear | 3 (3.33%) | 36 (6.02%) | 39 (5.67%) | 688 |
| Data are n (%) or mean (SD). SD – standard deviation. PPH – postpartum haemorrhage. | | | | |

71

|  | **Episiotomy** | | | |
| --- | --- | --- | --- | --- |
|  | **Yes** | **No** | **Total** | **Total N** |
|  | N=4,355 | N=10,713 | N=15,068 | 15,068 |
| Small-for-gestational age | 604 (13.89%) | 1,395 (13.06%) | 1,999 (13.30%) | 15,034 |
| Low birthweight (<2,500 g) | 620 (14.24%) | 2,072 (19.34%) | 2,692 (17.87%) | 15,066 |
| Preterm birth (<37 weeks) | 812 (18.65%) | 2,708 (25.28%) | 3,520 (23.36%) | 15,068 |
| Small vulnerable newborn * | 1,362 (31.32%) | 3,843 (35.96%) | 5,205 (34.62%) | 15,036 |
| Stillbirth ** | 161 (3.70%) | 875 (8.17%) | 1,036 (6.88%) | 15,068 |
| * Small-for-gestational age and/or low birthweight and/or preterm.(2) ** Both antepartum and intrapartum stillbirths. | | | | |

73

|  | **Nigeria** | **Pakistan** | **Tanzania** | **Zambia** | **Total** |
| --- | --- | --- | --- | --- | --- |
|  | **N=498** | **N=3,492** | **N=792** | **N=249** | **N=5,031** |
| Episiotomy | 319 (64.06%) | 2,855 (81.76%) | 137 (17.30%) | 73 (29.32%) | 3,384 (67.26%) |
| Maternal age (years) |  |  |  |  |  |
| Mean (SD) | 24.20 (4.41) | 23.66 (3.92) | 22.03 (3.53) | 21.06 (3.70) | 23.33 (3.99) |
| Gestational age (weeks) |  |  |  |  |  |
| Mean (SD) | 38.01 (2.36) | 37.35 (2.58) | 37.45 (2.93) | 35.84 (4.14) | 37.35 (2.74) |
| Anaemia severity at baseline |  |  |  |  |  |
| Moderate | 473 (94.98%) | 3,003 (86.00%) | 759 (95.83%) | 232 (93.17%) | 4,467 (88.79%) |
| Severe | 25 (5.02%) | 489 (14.00%) | 33 (4.17%) | 17 (6.83%) | 564 (11.21%) |
| Placental abnormalities | 1 (0.20%) | 72 (2.06%) | 16 (2.02%) | 4 (1.61%) | 93 (1.85%) |
| Hypertensive disorder | 24 (4.82%) | 257 (7.36%) | 78 (9.85%) | 27 (10.84%) | 386 (7.67%) |
| Birthweight to maternal height ratio |  |  |  |  |  |
| Mean (SD) | 11.50 (2.17) | 10.86 (2.13) | 11.34 (2.61) | 10.46 (3.12) | 10.98 (2.29) |
| Macrosomia (4,000g+) | 8 (1.61%) | 17 (0.49%) | 8 (1.01%) | 0 (0.00%) | 33 (0.66%) |
| Small vulnerable newborn | 177 (35.69%) | 1,304 (37.35%) | 299 (37.90%) | 145 (58.94%) | 1,925 (38.33%) |
| Labour induction | 67 (13.45%) | 580 (16.61%) | 42 (5.30%) | 24 (9.64%) | 713 (14.17%) |
| Labour augmentation | 237 (47.59%) | 1,265 (36.23%) | 303 (38.26%) | 63 (25.30%) | 1,868 (37.13%) |
| Instrumental delivery | 13 (2.61%) | 157 (4.50%) | 57 (7.20%) | 7 (2.81%) | 234 (4.65%) |
| Duration of labour (hours) |  |  |  |  |  |
| Mean (SD) | 13.79 (11.13) | 13.23 (14.29) | 18.10 (17.04) | 20.00 (26.09) | 14.38 (15.41) |
| Spontaneous perineal tear |  |  |  |  |  |
| No tear | 363 (72.89%) | 3,318 (95.02%) | 376 (47.47%) | 112 (44.98%) | 4,169 (82.87%) |
| Minor tear (1st/2nd degree) | 129 (25.90%) | 169 (4.84%) | 414 (52.27%) | 136 (54.62%) | 848 (16.86%) |
| Severe tear (3rd/4th degree) | 6 (1.20%) | 5 (0.14%) | 2 (0.25%) | 1 (0.40%) | 14 (0.28%) |
| Perineal sutures | 363 (72.89%) | 2,895 (82.90%) | 520 (65.66%) | 172 (69.08%) | 3,950 (78.51%) |
| Spontaneous cervical tear | 35 (7.03%) | 102 (2.92%) | 16 (2.02%) | 10 (4.02%) | 163 (3.24%) |
| Spontaneous vaginal tear | 24 (4.82%) | 50 (1.43%) | 8 (1.01%) | 17 (6.83%) | 99 (1.97%) |
| Data are n (%) or mean (SD). SD – standard deviation. PPH – postpartum haemorrhage. | | | | | |

75

77	before and after inverse probability of treatment weighting.

|  | **Before weighting** | | | **After weighting** | | |
| --- | --- | --- | --- | --- | --- | --- |
|  | **Mean** | | **Mean standardised difference** | **Mean** | | **Mean standardised difference** |
|  | **Episiotomy** | **No episiotomy** |  | **Episiotomy** | **No episiotomy** |  |
| Maternal age | 24.06 | 27.96 | 0.81 | 26.72 | 26.63 | 0.02 |
| Country | 1.99 | 2.18 | 0.33 | 2.15 | 2.13 | 0.04 |
| Gestational age | 1.82 | 1.77 | 0.11 | 1.76 | 1.79 | 0.05 |
| Anaemia severity | 0.12 | 0.14 | 0.07 | 0.16 | 0.13 | 0.09 |
| Haemoglobin at baseline | 83.15 | 82.86 | 0.02 | 82.30 | 82.99 | 0.06 |
| Primiparity | 0.77 | 0.17 | 1.53 | 0.34 | 0.35 | 0.03 |
| High parity | 0.00 | 0.03 | 0.22 | 0.04 | 0.02 | 0.09 |
| History of PPH | 0.00 | 0.01 | 0.10 | 0.02 | 0.01 | 0.07 |
| Placental abnormalities | 0.01 | 0.03 | 0.13 | 0.05 | 0.03 | 0.11 |
| Hypertensive disorder | 0.07 | 0.07 | 0.03 | 0.08 | 0.07 | 0.03 |
| Current long-term infection | 0.02 | 0.04 | 0.13 | 0.02 | 0.04 | 0.09 |
| Polyhydramnios | 0.01 | 0.01 | 0.01 | 0.01 | 0.01 | 0.00 |
| Uterine rupture | 0.00 | 0.00 | 0.02 | 0.00 | 0.00 | 0.02 |
| Birth weight | 2841.53 | 2827.49 | 0.03 | 2833.10 | 2835.80 | 0.00 |
| Birth weight to maternal height | 11.37 | 11.27 | 0.05 | 11.25 | 11.27 | 0.01 |
| Macrosomia | 0.01 | 0.02 | 0.05 | 0.03 | 0.02 | 0.04 |
| Small vulnerable newborn | 0.31 | 0.34 | 0.06 | 0.33 | 0.34 | 0.01 |
| Multiple gestation | 0.03 | 0.04 | 0.08 | 0.05 | 0.03 | 0.07 |
| Induction | 0.16 | 0.11 | 0.15 | 0.12 | 0.11 | 0.00 |
| Augmentation | 0.37 | 0.27 | 0.21 | 0.28 | 0.29 | 0.02 |
| Instrumental delivery | 0.04 | 0.02 | 0.13 | 0.02 | 0.03 | 0.04 |
| Pain control | 0.17 | 0.10 | 0.19 | 0.12 | 0.12 | 0.02 |
| Duration of labour | 13.74 | 12.59 | 0.08 | 13.13 | 12.88 | 0.02 |
| Spontaneous perineal tear | 0.09 | 0.14 | 0.15 | 0.13 | 0.13 | 0.02 |
| Spontaneous vaginal tear | 0.01 | 0.01 | 0.06 | 0.02 | 0.01 | 0.09 |
| Spontaneous cervical tear | 0.03 | 0.02 | 0.11 | 0.02 | 0.02 | 0.02 |
| PPH – postpartum haemorrhage. | | | | | | |

78

|  | **Scores within region of overlap** | **Scores outside region of overlap** | **Total *** |
| --- | --- | --- | --- |
|  | N=14,056 | N=892 | N=14,948 |
| Episiotomy | 4,230 (30.09%) | 103 (11.55%) | 4,333 (28.99%) |
| Maternal age (years) |  |  |  |
| Mean (SD) | 26.78 (5.32) | 33.68 (6.10) | 27.20 (5.61) |
| Country |  |  |  |
| Nigeria | 1,298 (9.23%) | 23 (2.58%) | 1,321 (8.84%) |
| Pakistan | 10,335 (73.53%) | 644 (72.20%) | 10,979 (73.45%) |
| Tanzania | 1,799 (12.80%) | 193 (21.64%) | 1,992 (13.33%) |
| Zambia | 624 (4.44%) | 32 (3.59%) | 656 (4.39%) |
| Gestational age (weeks) |  |  |  |
| Mean (SD) | 37.47 (2.55) | 36.09 (3.73) | 37.38 (2.66) |
| Anaemia severity at baseline |  |  |  |
| Moderate | 12,195 (86.76%) | 685 (76.79%) | 12,880 (86.17%) |
| Severe | 1,861 (13.24%) | 207 (23.21%) | 2,068 (13.83%) |
| Parity |  |  |  |
| 1 | 4,890 (34.79%) | 104 (11.66%) | 4,994 (33.41%) |
| 2-4 | 7,076 (50.34%) | 135 (15.13%) | 7,211 (48.24%) |
| 5-6 | 1,800 (12.81%) | 67 (7.51%) | 1,867 (12.49%) |
| 7+ | 290 (2.06%) | 586 (65.70%) | 876 (5.86%) |
| History of PPH | 122 (0.87%) | 27 (3.03%) | 149 (1.00%) |
| Placental abnormalities | 382 (2.72%) | 82 (9.19%) | 464 (3.10%) |
| Hypertensive disorder | 999 (7.11%) | 128 (14.35%) | 1,127 (7.54%) |
| Birthweight to maternal height ratio |  |  |  |
| Mean (SD) | 11.30 (2.28) | 9.87 (2.69) | 11.22 (2.33) |
| Macrosomia (4,000g+) | 226 (1.61%) | 7 (0.78%) | 233 (1.56%) |
| Small vulnerable newborn | 4,724 (33.61%) | 432 (48.43%) | 5,156 (34.49%) |
| Labour induction | 1,702 (12.11%) | 89 (9.98%) | 1,791 (11.98%) |
| Labour augmentation | 4,240 (30.17%) | 211 (23.65%) | 4,451 (29.78%) |
| Instrumental delivery | 321 (2.28%) | 98 (10.99%) | 419 (2.80%) |
| Duration of labour (hours) |  |  |  |
| Mean (SD) | 12.93 (14.76) | 12.13 (15.02) | 12.89 (14.77) |
| Spontaneous perineal tear |  |  |  |
| No tear | 12,301 (87.51%) | 882 (98.88%) | 13,183 (88.19%) |
| Minor tear (1st/2nd degree) | 1,737 (12.36%) | 10 (1.12%) | 1,747 (11.69%) |
| Severe tear (3rd/4th degree) | 18 (0.13%) | 0 (0.00%) | 18 (0.12%) |
| Perineal sutures | 5,590 (39.77%) | 110 (12.33%) | 5,700 (38.13%) |
| Spontaneous cervical tear | 312 (2.22%) | 20 (2.24%) | 332 (2.22%) |
| Spontaneous vaginal tear | 147 (1.05%) | 12 (1.35%) | 159 (1.06%) |
| Data are n (%) or mean (SD). SD – standard deviation. PPH – postpartum haemorrhage.  * Excludes 120 women with missing covariate or outcome data for the primary analysis of clinically diagnosed postpartum haemorrhage. | | | |

80

82 **haemorrhage (unadjusted and adjusted) (including women with propensity scores outside the region of**
83 **overlap)**

| Outcome | Number of women with outcome | | OR (95% CI) | |
| --- | --- | --- | --- | --- |
|  | Episiotomy | No episiotomy | Unadjusted | Adjusted |
| Clinically diagnosed postpartum haemorrhage | 358 / 4,333 (8.26%) | 670 / 10,615 (6.31%) | 1.37 (1.17 to 1.60) | 1.88 (1.33 to 2.65) |
| Calculated postpartum haemorrhage | 410 / 4,248 (9.65%) | 988 / 10,497 (9.41%) | 1.25 (1.07 to 1.46) | 1.61 (1.12 to 2.31) |
| Unadjusted and adjusted models include a random effect for hospital. Adjusted model also adjusts for confounders using inverse propensity score weighting. N=14,948 for clinically diagnosed postpartum haemorrhage. N=14,745 for calculated postpartum haemorrhage. OR; odds ratio. CI; confidence interval. | | | | |

84

86	representing shoulder dystocia on the clinically diagnosed postpartum haemorrhage odds ratio

| **Prevalence of shoulder dystocia (%)** | | **Odds ratio (95% CI)** | | |
| --- | --- | --- | --- | --- |
| **No episiotomy** | **Episiotomy** | **Effect of shoulder dystocia on PPH** | **Effect of episiotomy on clinical PPH** | **Effect of episiotomy on clinical PPH, correcting for residual confounding** |
| 1% | 2.0% | 1.5 | 1.88 (1.33 to 2.66) | 1.87 (1.32 to 2.65) |
| 1% | 2.0% | 2 |  | 1.86 (1.32 to 2.63) |
| 1% | 2.0% | 2.5 |  | 1.85 (1.31 to 2.62) |
| 1% | 3.5% | 1.5 |  | 1.86 (1.31 to 2.63) |
| 1% | 3.5% | 2 |  | 1.83 (1.30 to 2.60) |
| 1% | 3.5% | 2.5 |  | 1.81 (1.28 to 2.57) |
| 1% | 5.0% | 1.5 |  | 1.84 (1.30 to 2.61) |
| 1% | 5.0% | 2 |  | 1.81 (1.28 to 2.56) |
| 1% | 5.0% | 2.5 |  | 1.78 (1.26 to 2.51) |
| Prevalence of shoulder dystocia in women who do not receive an episiotomy was kept constant at 1%. Prevalence in women who receive an episiotomy was varied (2.0%, 3.5%, 5.0%). Effect of shoulder dystocia was also varied (OR=1.5, OR=2.0, OR=2.5). Vanderweele’s  methodology was used to correct the odds ratio (aOR=1.88 (1.33 to 2.66); table 3).^(4)^ | | | | |

87

88 **Table 14: Quantitative bias analysis examining the impact of a binary unmeasured confounder**
89 **representing shoulder dystocia on the calculated postpartum haemorrhage odds ratio**

| **Prevalence of shoulder dystocia (%)** | | **Odds ratio (95% CI)** | | |
| --- | --- | --- | --- | --- |
| **No episiotomy** | **Episiotomy** | **Effect of shoulder dystocia on PPH** | **Effect of episiotomy on calculated PPH** | **Effect of episiotomy on calculated PPH, correcting for residual confounding** |
| 1% | 2.0% | 1.5 | 1.63 (1.14 to 2.34) | 1.62 (1.13 to 2.33) |
| 1% | 2.0% | 2 |  | 1.61 (1.13 to 2.32) |
| 1% | 2.0% | 2.5 |  | 1.61 (1.12 to 2.31) |
| 1% | 3.5% | 1.5 |  | 1.61 (1.13 to 2.31) |
| 1% | 3.5% | 2 |  | 1.59 (1.11 to 2.28) |
| 1% | 3.5% | 2.5 |  | 1.57 (1.10 to 2.26) |
| 1% | 5.0% | 1.5 |  | 1.60 (1.12 to 2.29) |
| 1% | 5.0% | 2 |  | 1.57 (1.10 to 2.25) |
| 1% | 5.0% | 2.5 |  | 1.54 (1.08 to 2.21) |
| Prevalence of shoulder dystocia in women who do not receive an episiotomy was kept constant at 1%. Prevalence in women who receive an episiotomy was varied (2.0%, 3.5%, 5.0%). Effect of shoulder dystocia was also varied (OR=1.5, OR=2.0, OR=2.5). Vanderweele’s methodology was used to correct the odds ratio (aOR=1.63 (1.14 to 2.34); table 3).^(4)^ | | | | |

90

91 **Table 15: The association between episiotomy and cause-specific clinically diagnosed postpartum**
92 **haemorrhage (unadjusted and adjusted)**

| Outcome | Number of women with outcome | | OR (95% CI) | |
| --- | --- | --- | --- | --- |
|  | Episiotomy | No episiotomy | Unadjusted | Adjusted |
| Clinically diagnosed postpartum haemorrhage attributed to tearing | 2.96% (125 /  4,230) | 1.37% (135 /  9,826) | 2.57 (1.81 to  3.66) | 1.96 (1.09 to  3.52) |
| Clinically diagnosed postpartum haemorrhage attributed to uterine atony | 5.34% (226 /  4,230) | 4.53% (445 /  9,826) | 1.14 (0.97 to  1.34) | 1.62 (1.05 to  2.48) |
| Clinically diagnosed postpartum haemorrhage attributed to retained placenta | 1.37% (58 /  4,230) | 1.40% (138 /  9,826) | 1.01 (0.77 to  1.33) | 1.44 (0.86 to  2.40) |
| Unadjusted and adjusted models include a random effect for hospital. Adjusted model also adjusts for confounders using inverse propensity score weighting. N= 14,056. OR; odds ratio. CI; confidence interval. | | | | |

93

|  | **Before weighting** | | | **After weighting** | | |
| --- | --- | --- | --- | --- | --- | --- |
|  | **Mean** | | **Mean standardised difference** | **Mean** | | **Mean standardised difference** |
|  | **Episiotomy** | **No episiotomy** |  | **Episiotomy** | **No episiotomy** |  |
| Maternal age | 24.06 | 27.91 | 0.80 | 26.51 | 26.56 | 0.01 |
| Country | 1.99 | 2.19 | 0.35 | 2.14 | 2.13 | 0.01 |
| Gestational age | 1.83 | 1.79 | 0.10 | 1.78 | 1.80 | 0.06 |
| Haemoglobin at baseline | 86.01 | 86.54 | 0.07 | 86.15 | 86.46 | 0.04 |
| Primiparity | 0.77 | 0.17 | 1.51 | 0.36 | 0.36 | 0.00 |
| High parity | 0.00 | 0.03 | 0.23 | 0.01 | 0.02 | 0.07 |
| History of PPH | 0.00 | 0.01 | 0.09 | 0.02 | 0.01 | 0.10 |
| Placental abnormalities | 0.01 | 0.02 | 0.09 | 0.02 | 0.02 | 0.02 |
| Hypertensive disorder | 0.06 | 0.07 | 0.04 | 0.07 | 0.07 | 0.03 |
| Current long-term infection | 0.02 | 0.04 | 0.14 | 0.02 | 0.04 | 0.09 |
| Polyhydramnios | 0.01 | 0.01 | 0.00 | 0.01 | 0.00 | 0.01 |
| Uterine rupture | 0.00 | 0.00 | 0.02 | 0.00 | 0.00 | 0.02 |
| Birth weight | 2859.85 | 2858.16 | 0.00 | 2856.87 | 2858.11 | 0.00 |
| Birth weight to maternal height | 11.44 | 11.39 | 0.02 | 11.31 | 11.36 | 0.02 |
| Macrosomia | 0.01 | 0.02 | 0.05 | 0.03 | 0.02 | 0.06 |
| Small vulnerable newborn | 0.30 | 0.32 | 0.05 | 0.33 | 0.32 | 0.01 |
| Multiple gestation | 0.03 | 0.04 | 0.08 | 0.05 | 0.03 | 0.07 |
| Induction | 0.16 | 0.11 | 0.16 | 0.12 | 0.12 | 0.01 |
| Augmentation | 0.38 | 0.28 | 0.22 | 0.28 | 0.29 | 0.03 |
| Instrumental delivery | 0.04 | 0.02 | 0.13 | 0.02 | 0.02 | 0.03 |
| Pain control | 0.17 | 0.10 | 0.20 | 0.12 | 0.12 | 0.00 |
| Duration of labour | 14.02 | 12.91 | 0.08 | 13.18 | 13.22 | 0.00 |
| Spontaneous perineal tear | 0.09 | 0.15 | 0.17 | 0.14 | 0.14 | 0.00 |
| Spontaneous vaginal tear | 0.01 | 0.01 | 0.05 | 0.03 | 0.01 | 0.11 |
| Spontaneous cervical tear | 0.03 | 0.02 | 0.11 | 0.02 | 0.02 | 0.03 |
| PPH – postpartum haemorrhage. | | | | | | |

96

|  | **Before weighting** | | | **After weighting** | | |
| --- | --- | --- | --- | --- | --- | --- |
|  | **Mean** | | **Mean standardised difference** | **Mean** | | **Mean standardised difference** |
|  | **Episiotomy** | **No episiotomy** |  | **Episiotomy** | **No episiotomy** |  |
| Maternal age | 24.03 | 28.24 | 0.86 | 27.80 | 27.11 | 0.12 |
| Country | 2.02 | 2.10 | 0.20 | 2.19 | 2.08 | 0.21 |
| Gestational age | 1.72 | 1.66 | 0.13 | 1.69 | 1.68 | 0.02 |
| Haemoglobin at baseline | 61.46 | 60.13 | 0.17 | 62.61 | 60.34 | 0.30 |
| Primiparity | 0.76 | 0.12 | 1.66 | 0.23 | 0.29 | 0.14 |
| High parity | 0.01 | 0.03 | 0.13 | 0.15 | 0.02 | 0.47 |
| History of PPH | 0.00 | 0.01 | 0.17 | 0.00 | 0.01 | 0.15 |
| Placental abnormalities | 0.05 | 0.11 | 0.24 | 0.19 | 0.10 | 0.26 |
| Hypertensive disorder | 0.09 | 0.08 | 0.01 | 0.09 | 0.08 | 0.02 |
| Current long-term infection | 0.02 | 0.04 | 0.08 | 0.01 | 0.04 | 0.14 |
| Polyhydramnios | 0.00 | 0.01 | 0.07 | 0.00 | 0.01 | 0.08 |
| Uterine rupture | 0.00 | 0.00 | 0.03 | 0.00 | 0.00 | 0.00 |
| Birth weight | 2702.64 | 2637.84 | 0.12 | 2711.43 | 2689.84 | 0.04 |
| Birth weight to maternal height | 10.86 | 10.54 | 0.14 | 10.95 | 10.72 | 0.09 |
| Macrosomia | 0.01 | 0.01 | 0.04 | 0.01 | 0.02 | 0.08 |
| Small vulnerable newborn | 0.40 | 0.45 | 0.10 | 0.37 | 0.43 | 0.12 |
| Multiple gestation | 0.02 | 0.03 | 0.05 | 0.04 | 0.03 | 0.10 |
| Labour induction | 0.11 | 0.09 | 0.04 | 0.09 | 0.10 | 0.02 |
| Labour augmentation | 0.29 | 0.24 | 0.12 | 0.26 | 0.24 | 0.06 |
| Instrumental delivery | 0.05 | 0.02 | 0.16 | 0.02 | 0.04 | 0.08 |
| Pain control | 0.14 | 0.10 | 0.15 | 0.14 | 0.10 | 0.10 |
| Duration of labour | 11.58 | 10.60 | 0.07 | 12.89 | 10.68 | 0.13 |
| Spontaneous perineal tear | 0.06 | 0.07 | 0.03 | 0.12 | 0.07 | 0.16 |
| Spontaneous vaginal tear | 0.01 | 0.01 | 0.07 | 0.00 | 0.01 | 0.03 |
| Spontaneous cervical tear | 0.03 | 0.02 | 0.10 | 0.01 | 0.02 | 0.04 |
| PPH – postpartum haemorrhage. | | | | | | |

99

| Country | OR (95% CI) | |
| --- | --- | --- |
|  | Unadjusted | Adjusted |
| Nigeria | 1.68 (0.98 to 2.86) | 1.41 (0.79 to 2.55) |
| Pakistan | 1.18 (1.02 to 1.38) | 1.78 (1.12 to 2.82) |
| Tanzania | 1.70 (0.98 to 2.97) | 2.46 (1.21 to 5.01) |
| Zambia | 4.05 (1.96 to 8.38) | 3.37 (1.30 to 8.73) |
| Unadjusted and adjusted models include a random effect for hospital. Adjusted model also adjusts for confounders using inverse propensity score weighting. N= 14,056. OR; odds ratio. CI; confidence interval. | | |

102

105

106

107

108

109

Fractions)

| **Country** | **Episiotomy incidence in cohort (%)** | **Population Impact Fraction (%)** | | |
| --- | --- | --- | --- | --- |
|  |  | **Scenario 1** | **Scenario 2** | **Scenario 3** |
| Nigeria | 63% | 14% | 22% | 33% |
| Pakistan | 81% | 22% | 29% | 39% |
| Tanzania | 15% | N/a | N/a | 8% |
| Zambia | 29% | N/a | 3% | 17% |
| Scenario 1 – selective episiotomy incidence is 38% (mean incidence of episiotomy occurring in the selective arms of randomised trials). Scenario 2 – selective episiotomy incidence is 24% (lowest incidence of episiotomy occurring in the selective arms of randomised trials). Scenario 3 – selective episiotomy incidence is 5% (low population-level incidence of episiotomy).  All PIF calculations assume that episiotomy increases the risk of clinically diagnosed postpartum haemorrhage by 88% (main text; table 3). | | | | |

Table 20: Share of clinically diagnosed postpartum haemorrhage cases that could be prevented with selective episiotomy in multiparous women having non-instrumental births (Population Impact Fractions)

| **Country** | **Episiotomy incidence in cohort (%)** | **Population Impact Fraction (%)** | | |
| --- | --- | --- | --- | --- |
|  |  | **Scenario 1** | **Scenario 2** | **Scenario 3** |
| Nigeria | 6% | N/a | 4% | 4% |
| Pakistan | 11% | N/a | 8% | 8% |
| Tanzania | 2% | N/a | 0% | 1% |
| Zambia | 4% | N/a | 2% | 3% |
| Scenario 1 – selective episiotomy incidence is 26% (mean incidence of episiotomy occurring in the selective arms of randomised trials). Scenario 2 – selective episiotomy incidence is 2% (lowest incidence of episiotomy occurring in the selective arms of randomised trials). Scenario 3 – selective episiotomy incidence is 1% (low population-level incidence of episiotomy).  All PIF calculations assume that episiotomy increases the risk of clinically diagnosed postpartum haemorrhage by 88% (main text; table 3). | | | | |

110

111

112

113

114

115

116

117

118

119

Reference list

1. Khalil MR, Guldberg R, Nørgård BM, Uldbjerg N, Wehberg S. Child-Mother Index: a new risk factor for selected adverse maternal birth outcomes. AJOG Glob Reports. 2022;2(4).
2. Ashorn P, Ashorn U, Muthiani Y, Aboubaker S, Askari S, Bahl R, et al. Small vulnerable newborns—big potential for impact. Lancet. 2023;401(10389):1692–706.
3. Textor J, Zander B van der, Gilthorpe MK, Liskiewicz M, Ellison. GTH. Robust causal inference using directed acyclic graphs: the R package “dagitty.” Int J Epidemiol. 2016;45(6):1887–94.
4. Vanderweele TJ, Arah OA. Bias formulas for sensitivity analysis of unmeasured confounding for general outcomes, treatments, and confounders. Epidemiology. 2011;22(1):42–52.
