# Supplementary material for: Episiotomy and postpartum haemorrhage in women with moderate or severe anaemia: a cohort analysis of data from the WOMAN-2 trial
Source: Lancet Glob Health. Author manuscript; Available in PMC 2026 Oct 30. (PMC7619659; doi:10.1016/S2214-109X(25)00449-8)
Supplement: Supplementary file 7 [file EMS218565-supplement-Appendix_7.docx]

# Supplementary appendix 7

This Equitable Partnership Declaration (EPD) was submitted by the authors, and we reproduce it as supplied. It has not been peer reviewed. The Lancet’s editorial processes have not been applied to the EPD.

Supplement to: The WOMAN-2 Trial Collaborators. Episiotomy and postpartum haemorrhage in women with moderate or severe anaemia: a cohort analysis of data from the WOMAN-2 trial. Lancet Glob Health 2026; 14: e224–32.

## Equitable Partnership Declaration

### If any questions do not apply to your study, please indicate “N/A” for “not applicable.

For more information on how to complete this form see the Information for Authors document.

Researcher considerations

### 1. Please detail the involvement that researchers who are based in the country or countries of study had during a) study design; b) clinical study processes, such as processing blood samples, prescribing medication, or patient recruitment; c) data interpretation; and d) manuscript preparation, commenting on all aspects. If they were not involved in any of these aspects, please explain why.

This should include a thorough description of their leadership roles in the study. Are local researchers named in the author list or the acknowledgements, or are they not mentioned at all (and, if not, why)? Please also describe the involvement of early career researchers based in the location of the study. Some of this information might be repeated from the Contributors section in the manuscript. Note: we adhere to [ICMJE authorship criteria](https://www.icmje.org/recommendations/browse/roles-and-responsibilities/defining-the-role-of-authors-and-contributors.html) for naming authors on a paper.

**a) Study design:**

*This study is a cohort analysis of data collected for the WOMAN-2 clinical trial. Researchers in each of the study countries (Nigeria, Pakistan, Tanzania, Zambia) and the UK collaboratively developed the WOMAN-2 study design. This involved collecting adequate data to allow cohort*

*analyses such as this.*

**b) Clinical study processes:**

*Researchers based in the study countries contributed to the study processes, including developing the protocol, case-report forms and standard operating procedures, recruiting participants, collecting data and monitoring data accuracy. These activities were led by the*

*National Principal Investigators in each country.*

**c) Data interpretation:**

*Researchers based in the study countries contributed to interpreting the results of this analysis.*

**d) Manuscript preparation:**

*Researchers based in the study countries contributed to the manuscript preparation. We are submitting the manuscript under our group name ‘WOMAN-2 collaborators’. All co-author contributions are noted in the manuscript.*

### 2. How was funding used to remunerate and enhance the skills of researchers in the countries of study? And how was funding used to improve research infrastructure at the study sites?

Potentially effective investments into long-term skills and opportunities within local institutions could include training or mentorship in analytical techniques and manuscript writing, opportunities to lead all or specific aspects of the study, financial remuneration rather than requiring volunteers, and other professional development and educational opportunities.

Improvements to research infrastructure could include funding extended trial designs (eg, platform trials), establishment of long-term contracts for research staff, building research facilities, and setting up local control of funding allocation.

**Skills:**

*The funding for the WOMAN-2 trial enabled clinicians in the study countries to conduct large collaborative clinical trials that will improve their clinical care and increase their research skills.*

**Research infrastructure:**

*Although funding for the WOMAN-2 trial is short term, because we have managed to conduct many trials one after the other, we have long-term contracts for research staff and (rented) research facilities. There is considerable local control of funding allocation.*

### 3. How did you safeguard the researchers who implemented the study?

Please describe how you guaranteed safe working conditions for study staff, including provision of appropriate personal protective equipment, protection from violence, and prevention of overworking.

The study sponsor and research regulators visit and inspect the country-based trial co-ordinating centres and ensure safe working conditions. These settings are safe and clean and there is no risk of violence within the centres. We do not encourage overworking.

Benefits to the communities and regions of study

### 4. How does the study address the research and policy priorities of its location?

How were the local priorities determined and then used to inform the research question? Who decided which priorities to take forward? Which elements of the study address those priorities?

This cohort analysis examined the impact of episiotomy on postpartum haemorrhage in women with moderate or severe anaemia. Postpartum haemorrhage is the leading cause of maternal death in the study locations and anaemia is a major risk factor. Severe bleeding after birth and maternal anaemia are major health concerns for patients, doctors and policy makers everywhere in the world and are an obvious research priority. Our collaborative group (who have been working together for two decades) decided together to tackle this problem. We are not conducting research on maternal death for altruistic reasons. We are guided by the principle of solidarity. We work collaboratively with health professionals world-wide to conduct large multi-centre trials to improve outcomes in life threatening emergencies. We build global research partnerships to answer questions together that could not be answered by anyone working alone.

### 5. How will research products be shared in the community of study?

For instance, will you be providing written or oral layperson summaries for non-academic information sharing? Will study data be made available to institutions in the region(s) of study? The Lancet Global Health encourages authors to translate the summary (abstract) into relevant languages after paper editing; do you intend to translate your summary?

We routinely translate the abstracts of our papers and have been doing this for 20 years. The abstract of the current analysis has been translated into the major languages of each study country (Igbo, Hausa, Yoruba, Swahili, Urdu). To implement findings at the local level, these results are being discussed with clinical teams of participating hospitals. We also disseminate results via local social media as this has wider reach than written summaries. For example, we disseminated our previous trial findings via [Instagram](https://www.instagram.com/reel/DOK18fajM7V/?utm_source=ig_web_copy_link&igsh=cDE0b3M1ZTZnNGR5). We meet with Health Ministries to share our results. Our data sharing arrangement is described in the main manuscript.

### 6. How were individuals, communities, and environments protected from harm?

a) *How did you ensure that sensitive patient data were handled safely and respectfully? Was there any potential for stigma or discrimination against participants arising from any of the procedures or outcomes of the study?*

We collected anonymised data and followed the clinical trial regulations in all settings. It is highly unlikely that participants would have experienced stigma or discrimination from taking part in the study as they largely experienced usual clinical care.

b) *Might any of the tests be experienced as invasive or culturally insensitive?*

No. We used a standard point-of-care haemoglobin test that is not excessively invasive or culturally insensitive.

c) *How did you determine that work was sensitive to traditions, restrictions, and considerations of all cultural and religious groups in the study population?*

We work with local doctors. This study has examined episiotomy, which is part of usual clinical care in the study settings.

d) *Were biowaste and radioactive waste disposed of in accordance with local laws?*

N/A

e) *Were any structures built that would have impacted members of the community or the environment (such as handwashing facilities in a public space)? If so, how did you ensure that you had appropriate community buy-in?*

N/A

f) *How might the study have impacted existing health-care resources (such as staff workloads, use of equipment that is typically employed elsewhere, or reallocation of public funds)?*

We conduct simple pragmatic clinical trials that do not require any major changes from usual care. All additional work is paid for. The current cohort analysis has examined episiotomy, which is part of usual clinical care.

### 7. Confirm that local ethics review was sought, and please provide the approval number. If not sought, please explain why.

We have ethics committee approvals at all participating countries. The WOMAN-2 trial was approved by the London School of Hygiene & Tropical Medicine Research Ethics Committee (REF 15194) on May 10, 2018; the National Health Research Ethics Committee of Nigeria (NHREC/01/01/2007-29/09/2019) on Sept 29, 2019; the National Bioethics Committee of Pakistan on Nov 27, 2018 (NBC-340); the National Institute of Medical Research of Tanzania (NIMR/HQ/R.8a/Vol.IX/3767) on Aug 19, 2021; and the University of Zambia Biomedical Research Ethics Committee (REF 001-04-19) on Feb 2, 2019. Local site approvals were also obtained where relevant.

Secondary analyses

### 8. Have the data analysed in your study been extracted from another source, such as a national survey, rather than being directly collected by the authors of this paper?

No

### If the authors of this paper were not involved in data collection, how were the findings interpreted with sufficient contextual knowledge?

The Lancet Global Health *believe contextual understanding is crucial for informed data analysis and interpretation.*

N/A

### 9. Finally, please provide the title (eg, Dr/Prof, Mr/Mrs/Ms/Mx), name, and email address of an author who can be contacted about this statement.

**Name:** Dr Ian Roberts

**Email:** [**Ian.Roberts@lshtm.ac.uk**](mailto:Ian.Roberts@lshtm.ac.uk)
